# Low *PPP2R2A* expression promotes sensitivity to CHK1 inhibition in high-grade serous ovarian cancer

**DOI:** 10.7150/thno.96879

**Published:** 2024-11-04

**Authors:** Zhaojun Qiu, Deepika Singh, Yujie Liu, Chandra B. Prasad, Nichalos Bean, Chunhong Yan, Zaibo Li, Xiaoli Zhang, Goutham Narla, Analisa DiFeo, Qi-En Wang, Junran Zhang

**Affiliations:** 1 Department of Radiation Oncology, The James Comprehensive Cancer Center, The Ohio State University, Columbus, Ohio-43210, United States.; 2 Georgia Cancer Center, Augusta University Medical College, 1410 Laney Walker Blvd., CN-2134, Augusta, Georgia-30912, United States.; 3 Department of Pathology, The Ohio State University Wexner Medical Center, College of Medicine, Columbus, Ohio-43210, United States.; 4 Department of Biomedical Informatics, Wexner Medical Center, College of Medicine, The Ohio State University, Ohio-43210, United States.; 5 Department of Internal Medicine, University of Michigan, Ann Arbor, MI-48109, United States.; 6 The James Comprehensive Cancer Center, Pelotonia Institute for Immuno-Oncology, The Ohio State University, Columbus, Ohio-43210, United States.; 7 The James Comprehensive Cancer Center, Center for metabolism, The Ohio State University, Columbus, Ohio-43210, United States.

## Abstract

**Rationale:** High-grade serous ovarian cancer (HGSOC), the most lethal epithelial ovarian cancer subtype, faces persistent challenges despite advances in the therapeutic use of PARP inhibitors. Thus, innovative strategies are urgently needed to improve survival rates for this deadly disease. Checkpoint kinase 1 (CHK1) is pivotal in regulating cell survival during oncogene-induced replication stress (RS). While CHK1 inhibitors (CHK1i's) show promise as monotherapy for ovarian cancer, a crucial biomarker for effective stratification in clinical trials is lacking, hindering efficacy improvement and toxicity reduction. PP2A B55α, encoded by *PPP2R2A*, is a regulatory subunit of the serine/threonine protein phosphatase 2 (PP2A) that influences CHK1 sensitivity in non-small cell lung cancer (NSCLC). Given the complexity of PP2A B55α function in different types of cancer, here we sought to identify whether *PPP2R2A* deficiency enhances the sensitivity of HGSOC to CHK1 inhibition.

**Methods:** To determine whether PPP2R2A deficiency affects the sensitivity of HGSOC to CHK1 inhibition, we treated PPP2R2A knockdown (KD) HGSOC cells or HGSOC cells with naturally low PPP2R2A expression with a CHK1 inhibitor, then assessed cell growth in *in vitro* and *in vivo* assays. Additionally, we investigated the mechanisms contributing to the increased RS and the enhanced sensitivity to the CHK1 inhibitor in PPP2R2A-KD or deficient cells using various molecular biology assays, including western blotting, immunofluorescence, and DNA fiber assays.

**Results:** Our study suggests that *PPP2R2A-*KD elevates c-Myc-induced RS via upregulation of replication initiation, rendering HGSOC cells reliant on CHK1 for survival, including those resistant to PARP inhibitors.

**Conclusion:** Combined, these results identify *PPP2R2A*/PP2A B55α as a potential predictive biomarker for CHK1i sensitivity in HGSOC, as well as suggesting it as a therapeutic target to overcome PARP resistance.

## Introduction

Ovarian cancer stands as the most lethal gynecologic malignancy and ranks as the 5th leading cause of cancer-related deaths in women. A staggering 80% of patients with ovarian cancer are diagnosed at an advanced stage of disease, with 80% of these individuals experiencing recurrent disease despite aggressive surgical interventions and an initial high response rate to standard-of-care therapies, such as platinum/taxane-based chemotherapy 1-3. Notably, the emergence of drug resistance remains a challenge, even with advancements in the therapeutic use of Poly (ADP-ribose) polymerase (PARP) inhibitors for homologous recombination (HR)-deficient tumors, including ovarian cancer that is typically characterized as HR-deficient tumors 4. Epithelial ovarian cancer (EOC), which constitutes >90% of cases of ovarian cancer, especially the high-grade serous ovarian cancer (HGSOC) subtype, contributes significantly to most ovarian cancer-related deaths 5, 6. The 5-year survival rate for late stage EOC hovers around 30%, necessitating the urgent need for novel treatment strategies for HGSOC.

Oncogenes and tumor suppressor deficiencies often drive ovarian cancer, with distinct genetic profiles in various subtypes. While low-grade serous ovarian cancers exhibit high frequencies of KRAS Proto-Oncogene, GTPase (KRAS) and B-Raf Proto-Oncogene, Serine/Threonine Kinase (BRAF) oncogene mutations, HGSOC is characterized by a prevalence of p53 and tumor suppressor mutations alongside the absence of KRAS/BRAF mutations 7. Although oncogenes can be directly targeted due to their upregulated activity, tumor suppressor gene products cannot be directly targeted because of their deficiency. Therefore, the identification of the specific targetable pathway(s) that are altered in HGSOC because of deficiency in a recurrently altered tumor suppressor gene could provide an opportunity to reveal potential new therapeutic approaches.

The Ataxia-telangiectasia-mutated-and-Rad3-related kinase (ATR) and its downstream effector checkpoint kinase 1 (CHK1) play pivotal roles in the replication stress (RS) response, a branch of the DNA damage response (DDR) implicated in managing interferences and DNA damages during replication. RS can be triggered by exogenous agents, as well as by the endogenous deregulation of oncogenes, such as c-MYC Proto-Oncogene, BHLH Transcription Factor (Myc), Ras and cyclin E 8. ATR-CHK1 activation mitigates RS stress by orchestrating downstream events, making cancer cells with oncogene-induced RS particularly reliant on this pathway for survival 9-18. Despite promising findings in preclinical studies, the clinical benefits of ATR and CHK1 inhibitors are limited by their toxicities 19-22. The identifying biomarkers that are predictive of their responsiveness and understanding the conditions leading to RS in cancer cells would allow for an enhanced efficacy of these inhibitors, possibly at doses that are not unduly toxic.

Protein phosphatase 2 (PP2A), a family of heterotrimeric holoenzymes, accounts for the majority of serine/threonine phosphatase activity in human cells 23-25. PP2A comprises one catalytic subunit (C), one scaffolding subunit (A) and one regulatory subunit 25. The PP2A regulatory subunits are classified into four distinct families, B/PR55, B'/PR61, B”/PR72 and PTP/PR53, with each family containing at least four members 23. Thus, the specific function and substrate specificity of each PP2A holoenzyme is determined by the regulatory subunit bound to the PP2A AC dimer 23. The roles of PP2A in oncogenic transformation and cancer therapy remains incompletely understood and are also conflicting as outlined below. In aggregate, PP2A has been suggested to act as a tumor suppressor, based on loss-of-function analysis using PP2A catalytic inhibitors (*e*.*g*., okadaic acid) or viral oncoproteins that suppress PP2A activity 26-30. In support of this concept, the specific subunits of PP2A have been shown to be frequently mutated or deleted in cancers 31, 32. Deletions and mutations of *PPP2R2A*, the gene that encodes PP2A B55α, a protein involved in numerous human cancer types, including breast, prostate, primary plasma leukemia, acute myeloid leukemia and ovarian cell carcinoma, has been observed. For instance, 57.14% of ovarian cancers have reduced expression of *PPP2R2A* with most cases caused by *PPP2R2A* loss of heterozygosity 31. In addition, heterozygous and homozygous deletions of *PPP2R2A* correlate with the loss of the *PPP2R2A* transcript in estrogen receptor-positive luminal B breast cancer 32. Somatic deletions of *PPP2R2A* are also detected in 67% of prostate tumor samples 33, and primary plasma cell leukemia 34. Furthermore, loss-of-function mutations in *PPP2R2A* were also observed in acute leukemia blasts 35. However, despite most studies suggesting its tumor suppressor role, several others indicate that PP2A can promote the activation of oncogenic signaling pathways when associated with specific regulatory subunits 36. For example, it has been demonstrated that PPP2R2A promotes tumorigenesis and metastasis in pancreatic cancer cells 37 but inhibits metastasis in lung cancer 38. In addition to the conflicting data regarding the role of PP2A B55α in tumorigenesis and metastasis, the impact of *PPP2R2A* deficiency on cancer therapy is varied as well. *PPP2R2A* knockdown leads to increased sensitivity to PARP inhibition in lung cancer 31. We previously utilized a genome-wide loss-of-function screen and found that reduced expression of B55α increased cellular sensitivity to CHKi's in non-small cell lung cancer (NSCLC) because of increased RS due to upregulation of oncogenic c-Myc expression 39. Interestingly, *PPP2R2A* downregulation is also involved in microRNA-221-mediated cisplatin resistance in osteosarcoma cells, linking *PPP2R2A* low expression to cisplatin resistance 40. Recently, we found that PPP2R2A low expressing NSCLC cells also display cisplatin resistance 38. Further, loss-of-function mutations in *PPP2R2A* and the disappearance of B55α expression were associated with increased sensitivity to an Akt inhibitor in acute leukemia blasts, but less responsiveness to a PP2A activator 35. Therefore, the impact of B55α low expression/*PPP2R2A* deficiency varies among different cancer types and the anti-tumor drugs used. These findings highlight the need to explore the specific impact of PP2A B55α expression/*PPP2RA* deficiency on each cancer type for optimal clinical trial design.

Here, we explored the impact of *PPP2R2A* KD/deficiency on CHK1 inhibitor sensitivity in HGSOC cells. We found that PP2A B55α low expression/*PPP2R2A* deficiency increased the sensitivity to CHK1 inhibition in HGSOC cells, even in cells with PARP inhibitor resistance. Mechanistically, c-Myc activity is implicated in *PPP2R2A* deficiency-induced alterations of replication initiation, RS and sensitivity to CHK1 inhibitors. Consequently, our study suggests that PP2A B55α low expression/*PPP2R2A* deficiency predicts the response to CHK1 inhibition in treating HGSOC cells, presenting a promising avenue for improving therapeutic outcomes in this challenging malignancy.

## Materials and Methods

### Bioinformatics analysis

Data regarding *PPP2R2A* expression and mutations in human ovarian cancer patients were sourced from three TCGA studies from cBioportal (Nature 2011, *n* = 489; Firehose Legacy, *n* = 617; PanCancer Atlas, *n* = 585.). Overall survival analysis of ovarian cancer patients featuring the *PPP2R2A* probe 202313 was conducted using PrognoScan (http://dna00.bio.kyutech.ac.jp/PrognoScan/index.html). Additionally, overall survival and progression-free survival data based on *PPP2R2A* expression (probe 228013) were obtained from Kmplot (https://kmplot.com/). All the bioinformatics analysis were conducted by August 1, 2023.

### Cell lines, viruses, plasmids and inhibitors

OVCAR3, PEO1 and PEO4 cells were cultured in RPMI1640 medium (Hyclone), CAOV3 and HEK-293T cells were cultured in DMEM medium (Hyclone), OV90 cells were cultured in 1:1 mixture of MCDB 105 medium (Cell Applications) and Medium 199 (Hyclone). The media for all experiments were supplemented with 10% fetal bovine serum (Gibco) and maintained in a humidified atmosphere with 5% CO2 at 37 °C. Cells that had undergone ten or fewer passages were utilized for the experiments. All the ovarian cancer cells were gifts from Dr. Qi-En Wang (The Ohio State University). All cells underwent STR profiling by the MCIC Genomics core at The Ohio State University in 2024 to ensure authentication. Additionally, the absence of Mycoplasma contamination was confirmed in 2024 using the LookOut® Mycoplasma PCR Detection Kit (MP0035, Sigma) for all cell lines.

All shRNAs were purchased from Sigma-Aldrich. The specific shRNAs used include shPPP2R2A-1 (TRCN0000002490), shPPP2R2A-2 (TRCN0000002491), shPPP2R5A-1 (TRCN0000039618), shPPP2R5A-2 (TRCN0000039622), shCHK1-2 (TRCN0000000500), and shCHK1-3 (TRCN0000000502). For lentivirus packaging, pCMV delta R8.2 (Addgene, Plasmid #12263) and pCMV-VSV-G (Addgene, Plasmid #8454) were used. The lentiviruses were packaged in HEK-293T cells. pBABEpuro/c-Myc and pBABEpuro/c-MycS62A plasmids were generously provided by Dr. Peter J. Hurlin from Oregon Health and Science University. pUMVC (Addgene, Plasmid #8449) and pCMV-VSV-G (Addgene, #8454) were used for retrovirus packaging. The adenoviruses were packaged in HEK-293T cells.

The cells were transduced with a mixture of lentivirus or adenovirus and polybrene (Sigma-Aldrich, TR-1003-G). Eight h after transduction, the supernatant was replaced with fresh cell culture medium. Forty-eight h later, the cells were screened with puromycin (Thermo Fisher, A11138-03) to eliminate non-transduced cells, resulting in the generation of stable knockdown or overexpression cells.

The CHK1 inhibitor LY2603618 (A8638) and the PARP inhibitor ABT-888 (A3002) were purchased from APExBIO Technology, and the PARP inhibitor AZD2281 (S1060) and the c-Myc inhibitor 10058-F4 (S7153) were procured from Selleckchem.

### MTT assays

MTT assays were conducted following established protocols, as previously described 39.

### Real-Time Quantitative Reverse Transcription PCR (qRT-PCR)

Total RNAs were extracted using the RNeasy Mini Kit (Cat: 74016, Qiagen) and cDNA was synthesized from 1 μg of purified total RNA using the Transcriptor First Strand cDNA Synthesis Kit (Cat:4897030001, Roche) ushing oligo dT primers. qRT-PCR was conducted as described in our previous publications 39. The following primers were used for qRT-PCR: GAPDH forward/reverse primers: 5'-CTCTGCTCCTCCTGTTCGAC-3'/5'-TTAAAAGCAGCCCTGGTGAC-3'; PPP2R2A forward/reverse primers: 5'-CCACCTTTATCTCCTGTTGC-3'/5'-TTTCTCAGGTGAAAGGAGCAG-3'; c-Myc forward/reverse primers: 5'-AAAGGCCCCCAAGGTAGTTA-3'/5'-GCACAAGAGT TCCGTAGCTG-3'.

### Immunofluorescence assays

Immunofluorescence assays were carried out as described previously 39. The primary antibodies used for immunofluorescence were γH2AX (JBW301, 1:500, Millipore) and p-RPA2 (S32) (A300-246A, 1:500, Bethyl). Goat anti-rabbit IgG (H+L) Alexa Fluor 594 secondary antibody (A-11012, 1:400, Thermo Fisher Scientific) and goat anti-mouse IgG (H+L) Alexa Fluor 488 secondary antibody (A-28175, 1:400, Thermo Fisher Scientific) were used for immunofluorescence assays.

To quantify the immunofluorescence images in their TIFF format we utilized ImageJ software (NIH). Background fluorescence was subtracted using the Subtract Background tool. Regions of interest (ROIs) were selected using the Rectangle tools. Fluorescence intensity within each ROI was measured (Analyze > Measure), and mean intensity values were recorded. Data analyses were conducted by GraphPad Prism software.

### Comet assay

The Neutral Comet Assays were conducted using the Comet Assay kit (#4250-050-K, Trevigen), following the manufacturer's instructions. Analysis of comets was performed using TriTek CometScore software ver. 2.0.0.38.

### Western blotting

Immunoblotting was performed as previously described 10, 41. For chromatin CDC45 isolation, 1 × 10^6^ OVCAR3 cells were suspended in 100 μL of buffer A [10 mmol/L HEPES (pH 7.9), 10 mmol/L KCl, 1.5 mmol/L MgCl2, 0.34 mol/L sucrose, 10% glycerol, 1 mmol/L dithiothreitol, and a protease inhibitor mixture (Roche Molecular Biochemicals)]. Triton X-100 was then added to achieve a final concentration of 0.1%, and the cells were incubated for 10 min on ice. Nuclei were collected in the pellet (P1) through low-speed centrifugation (1,500 × *g*, 4 min, 4 °C). The nuclei (P1) were washed once with buffer A and subsequently lysed in 200 μL of buffer B (3 mmol/L EDTA, 0.2 mmol/L EGTA, 1 mmol/L DTT, and a protease inhibitor mixture). After a 10-min incubation on ice, soluble nuclear proteins (S2) were isolated from chromatin by centrifugation (2,000 × *g*, 4 min). The insoluble chromatin (P2) underwent a single wash in buffer B and was centrifuged again under the same conditions. The final chromatin pellet (P3) was resuspended and boiled in 30 μL of 1x Laemmli buffer. The primary antibodies used for western blot were PP2A B55α (PPP2R2A, #5689, 1:1000, Cell Signaling Technology); c-Myc (SC-40, 1:500, Santa Cruz Technology); phospho-c-Myc Ser62 (#13748, 1:500; Cell Signaling Technology); phospho-c-Myc Thr58 (Y011034, 1:1000; Applied Biological Materials Inc.); phospho-RPA2 Ser33 (A300-246A, 1: 1000, Bethyl); RPA2 (Clone NA18, 1:100, Calbiochem/EMD Millipore); β-Actin (Clone AC-74, 1:50000, Sigma-Aldrich); CHK1 (G-4, 1:200, Santa Cruz Technology); phospho-CHK1 antibody Ser345 (#133D3,1:500, Cell Signaling Technology); CDC45 (G-12 sc55569, 1:200, Santa Cruz Technology); γH2AX (ser139, clone JBC301, 1:500, Millipore); H2AX (#7631, 1:1000, Cell Signaling Technology); Histone H3 (#9715, 1:1000, Cell Signaling Technology); PP2A B56α (PPP2R5A, ab89621,1:1000, Abcam); CHK2 (#6334,1:1000, Cell Signaling Technology); phospho-CHK2 antibody T68 (#2197,1:1000, Cell Signaling Technology); Cyclin E1 (#20808, 1:1000, Cell Signaling Technology). The following secondary antibodies were used for Western blotting: goat-anti-mouse IgG-horseradish peroxidase (HRP) conjugated (#7076S, 1:1000, Cell Signaling Technology), goat-anti-rabbit IgG-HRP conjugated (#7074S, 1:1000, Cell Signaling Technology), and goat anti-rat IgG-HRP conjugated (#7077S, 1:1000, Cell Signaling Technology).

Western blot images were quantified using ImageJ software. We used the Rectangle tool to select each band. Regions of interest (ROIs) were defined for each band and analyzed for pixel intensity using the gel analysis function. Density values of each protein were obtained and normalized against the loading control β-Actin for comparison. Data analyses were performed using GraphPad Prism software.

### BrdU incorporation and cell cycle analysis

OVCAR3 cells were incubated with 10 µM 5-Bromo-2-deoxyuridine (BrdU) (Biolegend, #370307) for 1 h, after which the medium was replaced with fresh medium. The cells were then harvested and processed according to the manufacturer's protocol.

### DNA fiber assays

DNA fiber assays were performed following established protocols as previously described 39. The antibodies used for the DNA fiber assay were BrdU antibody (#347580, 1:20, BD Biosciences) and CldU antibody (ab6326, 1:100, Abcam). Alexa goat anti-mouse 594 (A-11005, 1:200, Invitrogen) and Alexa goat anti-rat 488 (A-11006, 1:200, Invitrogen) were used as secondary antibodies.

### Microscopy

The images from the comet assay and DNA fiber assays were observed at 60× magnification using a Zeiss Axio Observer inverted fluorescence microscope (X-Cite 120LED). Representative images for immunofluorescence assays were captured using a Zeiss LSM510 Meta confocal microscope.

### Cycloheximide assay

Cycloheximide assays were conducted as previously described 39

### Xenograft studies

Female athymic nude (NCr-nu/nu) mice, aged of 4-5 weeks, were obtained from the athymic nude mouse colony maintained by the Target Validation Shared Resource (TVSR) at The Ohio State University. The original breeders (strain #553 and #554) for the colony were received from the NCI Frederick facility/Charles River. Xenografts were initiated by subcutaneous injections of OVCAR3 cells (5 × 10^6^ cells) into right flank of the mice. Tumor diameters were measured with a digital caliper, and the tumor volumes were calculated using the following formula: Volume = (width)^2^ × length/2. Once tumor volume reached ~100 mm^3^, the mice were subjected to treatment with vehicle control DMSO or a CHK1 inhibitor (25 mg/kg of LY2603618) via intraperitoneal injection twice a day for 3 days, followed by 4 days of rest. All mice were housed under barrier conditions, and the experiments followed approved protocols and conditions set by the Institutional Animal Care and Use Committee (IACUC) of The Ohio State University.

## Results

### *PPP2R2A* knockdown (KD) sensitizes HGSOC cells to CHK1 inhibition *in vitro*

In support of the role of PP2A B55α as a tumor suppressor in ovarian cancer 42**,** 57.14% of ovarian cancer shown *PPP2R2A* expression reduction due to 41.75% of ovarian cancer samples with *PPP2R2A* loss of heterozygosity (LOH) 31, 43. According to the analysis of three TCGA data sets from cBioportal, we found that *PPP2R2A* expression is lower or deeply deleted in approximately 30% of ovarian cancer (Figure S1A). By Kaplan-Meier survival analyses of the HGSOC data we found that low expression of *PPP2R2A* determined by microarray correlates with decreased patient survival (Figure S1B-D). Thus, *PPP2R2A* expression is frequently downregulated in ovarian cancer, which is strongly associated with a poor prognosis. Targeting ovarian cancer cells with a deficiency in *PPP2R2A* presents an unmet clinical need.

**Figure 1:**
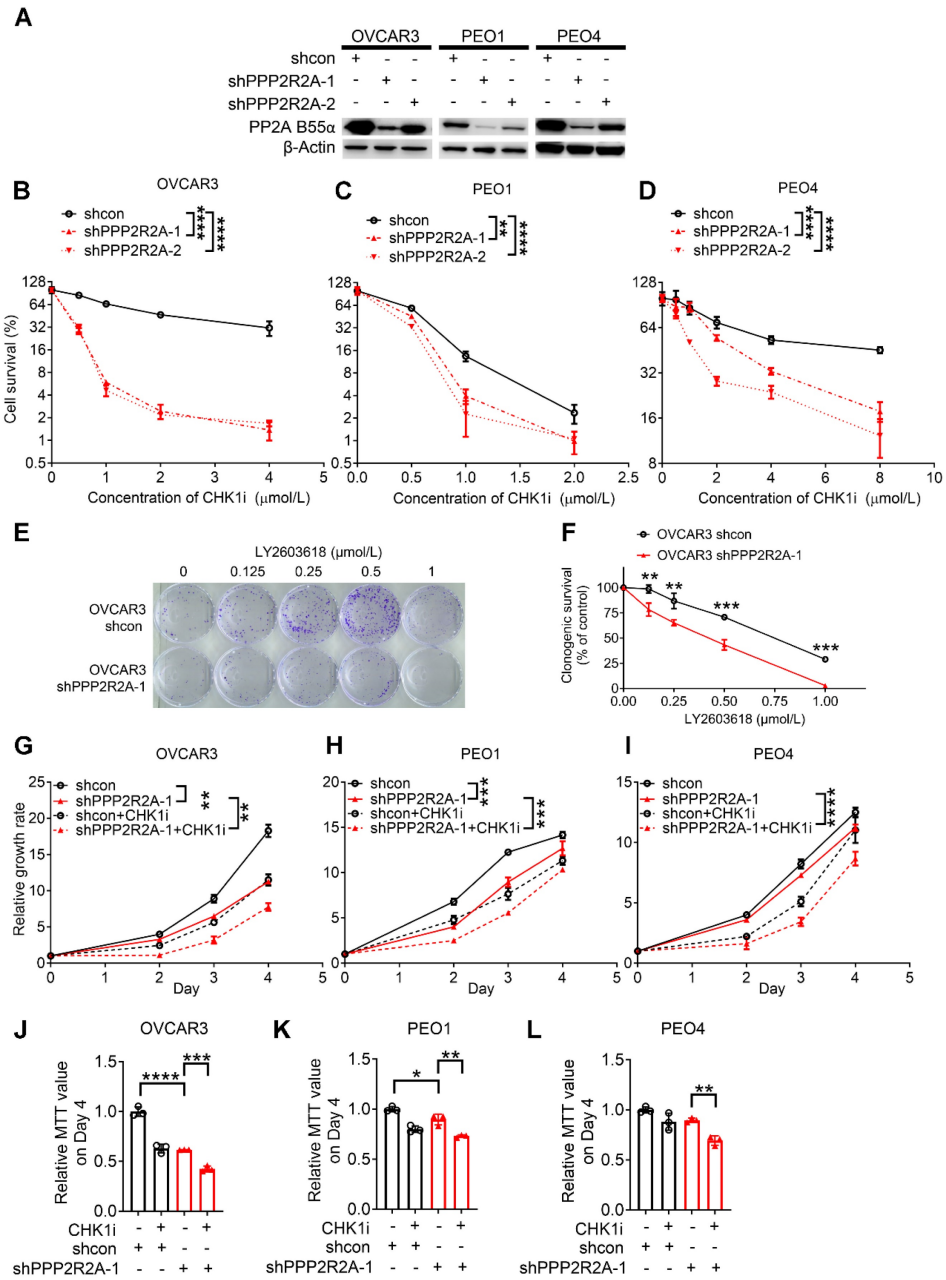
***PPP2R2A* deficiency is synthetically lethal with CHK1 inhibition *in vitro****.* (**A**) Protein expression of B55α in the three indicated ovarian cancer cell lines upon *PPP2R2A* knockdown (KD). (**B-D**) Cell survival based on cellular toxicity assays upon *PPP2R2A* KD and treatment with a CHK1i for 48 h at different doses OVCAR3 (**B**), PEO4 (**C**) and PEO1 (**D**) cells. *n*=3, biological repeats. (**E-F**) Colony formation assays of control and *PPP2R2A* KD HGSOC cells with CHK1 inhibition. OVCAR3 cells were treated with 1 µmol/L of CHK1 inhibitor LY2603618 for 1 day, followed by incubation in fresh medium for an additional 9 days. Representative figures from OVCAR3 cells are shown in (**E**) and statistical analysis results are shown in (**F**). *n*=3, biological repeats. (**G-L**) Relative cell growth rates of OVCAR3 (**G**), PEO1 (**H**) and PEO4 (**I**) cells or their MTT values on day 4 (**J-L**) upon CHK1 inhibition (1 µmol/L for OVCAR3 and PEO4, 0.5µmol/L for PEO1) and/or *PPP2R2A* KD. *n*=3, biological repeats. *, *P* < 0.05, **, *P* < 0.01, ***, *P* < 0.001, ****, *P* < 0.0001, two-way ANOVA, followed by Bonferroni post hoc analysis for multiple comparisons was used to determine statistical significance in (**B-D**, **G-I**); Statistical significance in (**F**, **J-L**) was determined by one-way ANOVA, followed by Bonferroni post hoc analysis for multiple comparisons.

We have previously reported that *PPP2R2A* KD is synthetically lethal with the CHK1 inhibitor (CHKi) LY2603618 in NSCLC 39. To determine if PPP2R2A deficiency also affects the potency of a CHK1 inhibitor in HGSOC, we first determined the impact of *PPP2R2A* KD on the sensitivity to CHK1 inhibition in the three HGSOC cell lines, PEO1 and PEO4 and OVCAR3. PEO1 and PEO4 cells are isogenic with a difference in the status of BRCA2 (Breast And Ovarian Cancer Susceptibility Protein 2), a HR protein. Both PEO1 and PEO4 cells harbor a BRCA2 mutation. However, PEO1 is an HR-deficient cell line and is sensitive to PARP inhibition, whereas PEO4 cells is HR proficient and is resistant to PARP inhibition due to a secondary mutation that restores the function of BRCA2 44. We first generated the HGSOC cells with stable *PPP2R2A* KD (Figure 1A). By cellular toxicity assays we identified that *PPP2R2A* KD sensitized the three cell lines to CHK1 inhibition, leading to a significant increase in toxicity and cell death in a dose-dependent manner (Figure 1B-D). This result was also validated by clonogenic assays in OVCAR3 cells using a second different shRNA targeting different regions of PPP2R2A (Figure 1E-F, Figure S2A-B). Similarly, the three HGSOC cells lines that were depleted of *PPP2R2A* expression by shRNAs demonstrated a significant suppression of cellular proliferation upon treatment with a CHK1i for 24 h (Figure 1G-L, Figure S2C-H). To verify the specificity of the antitumor activity of CHKi, we next determined the impact of CHK inhibition by KD via shRNAs. CHK1 KD (Figure S3A-B) leads to slow cell growth of OVCAR3 cells. This effect was significantly enhanced in cells with concurrent *PPP2R2A* KD (Figure S3C-F), which is the same as what we observed in cells treated with the CHK1i.

Given that PARP inhibition is a standard therapy for treating HGSOC in the clinic, we next also determined the sensitivity of the cell lines to a PARP inhibitor using cellular toxicity assay. Notably, *PPP2R2A* KD increased the sensitivity to PARP inhibition in the PEO1 cell line (Figure S4A-B). No significant impact was found regarding PAPR inhibitor resistance in the PEO4 cell line (Figure S4C-D), although it has suggested that PPP2R2A KD leads to increased sensitivity to PARPi in NSCLC 31. Thus, CHK1 inhibition is more effective in targeting HGSOC cells with PP2A B55α low expression, including the HGSOC cells with PARP inhibitor resistance. However, *PPP2R2A* KD has no impact on the sensitivity to a PARP inhibitor in the PARP inhibitor-resistant cells.

To further validate our results, we next compared the CHK1 inhibitor sensitivity among the cell lines with relatively low and high PP2A B55α levels. PEO1, PEO4 and OVCAR3 cells have relatively high expression levels of PP2A B55α, whereas CAOV3 and OV90 cells display relatively low or barely any PP2A B55α expression, respectively (Figure S5A). PEO1 cells demonstrate sensitivity to both CHK1 and PARP inhibitors compared to CAOV3 and OV90 cells (Figure S5B-C) because this cell line is characterized by a defective BRCA2, a key protein required for HR 45. For the other four cell lines, HGSOC cells with relatively low levels of PP2A B55α (CAOV3 and OV90) are more sensitive to treatment with the CHK1 inhibitor LY2603618 in a dose-dependent manner, as detected by MTT assays (Figure S5B), compared to the cell lines with relatively high PP2A B55α expression (PEO4 and OVCAR3). Furthermore, CHK1 inhibition resulted in a slower growth rate of OV90 and CAOV3 cells (Figure S5D-E), which have a relatively low expression of *PPP2R2A*, but it did not affect the proliferation of OVCAR3 (Figure S5F), PEO1 (Figure S5G), and PEO4 (Figure S5H) cells, which have relatively high expression of *PPP2R2A*. In summary, our results suggest that low PP2A B55α expression is associated with a greater sensitivity to CHK1 inhibition, even for HGSOC cells with resistance to PARP inhibition.

### *PPP2R2A* KD sensitizes OVCAR3 cells to CHK1 inhibition *in vivo*

**Figure 2:**
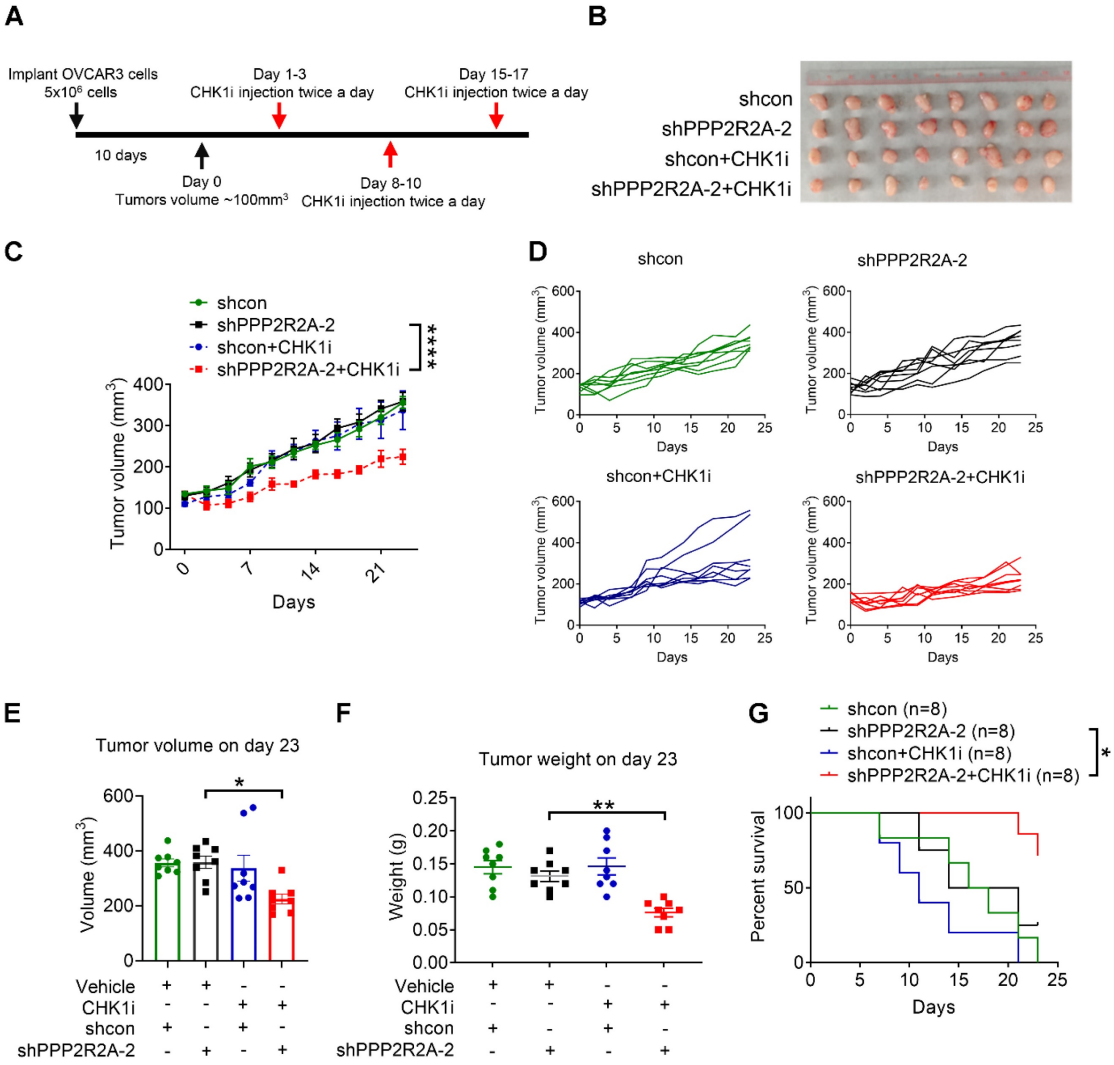
***PPP2R2A* KD sensitizes OVCAR3 cells to CHK1 inhibitors *in vivo****.* (**A**) A schematic diagram illustrating the experimental regimen. Female nude mice were subcutaneously inoculated with 5 x 10^6^ OVCAR3 cells carrying shcon or shPPPP2R2A-2. When the tumor volume reached ~100 mm^3^, mice were then randomized to four groups and treated with vehicle or CHK1 inhibitor LY2603618 via intraperitoneal injection twice a day for three days followed by 4 days of rest for three cycles. (**B-G**) The effects of CHK1i on tumor growth of OVCAR3 cells with stable *PPP2R2A* KD. The gross morphology of the xenograft tumors for each group on day 23 is shown in (**B**). CHK1 inhibition led to tumor volume reduction in *PPP2R2A* KD tumors (**C**). ****, *P* < 0.0001, two-way ANOVA, followed by Bonferroni post hoc analysis for multiple comparisons was used to determine statistical significance. Individual tumor growth curves over time for each group (**D**). *n*=8, number of tumors in **B**-**G**. Quantification of tumor volume (**E**) or tumor weight (**F**) on day 23. *, *P* < 0.05, **, *P* < 0.01, statistical significance was determined by one-way ANOVA, followed by Bonferroni post hoc analysis for multiple comparisons. CHK1 inhibition increases the survival of PPP2R2A defective tumors. Kaplan-Meier survival curves of different treatment groups and significance were determined by Mantel-Cox test (*, *P* < 0.05) (**G**).

To further validate our *in vitro* observations, we performed an *in vivo* assay utilizing a xenograft model of OVCAR3 as only this line has been reported to successfully form tumors in nude mice upon subcutaneous implantation 46. We initiated CHK1 inhibitor treatment once the tumor reached a volume approximately of 100 mm^3^ (Figure 2A). Animals harboring xenograft tumors derived from *PPP2R2A* stable KD cells exhibited a substantial reduction in tumor size upon CHK1i treatment, resulting in a marked inhibition of tumor growth (Figure 2B-D). The combination group exhibited significantly smaller tumor volume and weight compared to the group with *PPP2R2A* KD alone at the end of treatment (Figure 2E-F). Furthermore, this notable decrease of tumor growth correlated with prolonged overall survival, particularly in animals with tumors originating from *PPP2R2A*-depleted cells treated with CHK1 inhibition (Figure 2G). In conclusion, the *in vivo* results indicate that PPP2R2A KD significantly increases the sensitivity of the tumors to the effects of the CHK1i.

### Inhibition of CHK1 results in elevated RS, especially in HGSOC cells with *PPP2R2A* KD

**Figure 3:**
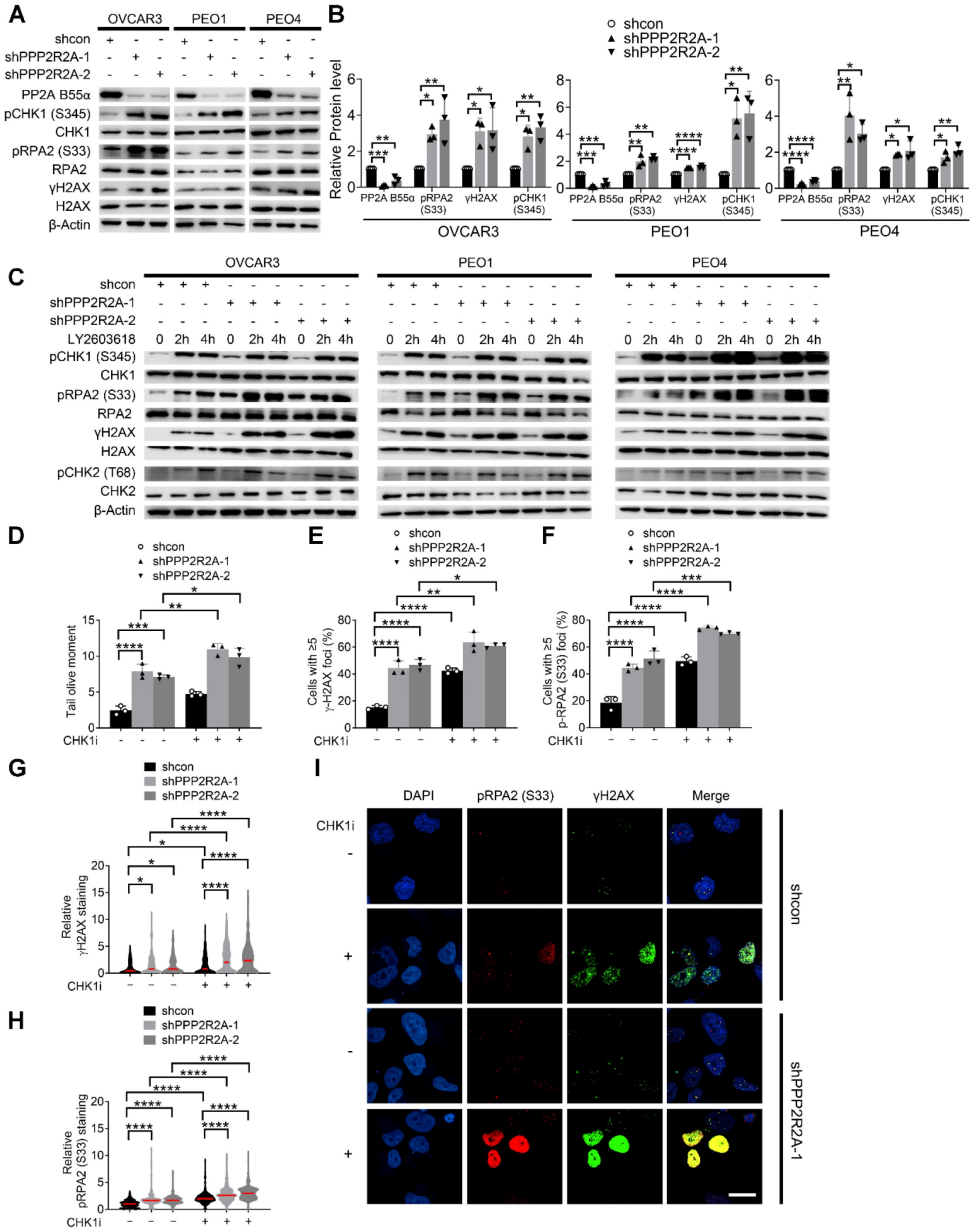
**CHK1 inhibition leads to the increased RS, particularly in *PPP2R2A* KD HGSOC cells.** (**A**,** B**) Expression of the indicated RS markers in *PPP2R2A* KD ovarian cancer cells**.** Western blot of RS markers (**A**), statical analysis of three independent assays (**B**). *n* = 3 in **B**, biological repeats. (**C**) Expression of pRPA2 S33, pCHK1 and γH2AX after 2 h or 4 h of CHK1 inhibitor treatment (1 µmol/L of LY2603618) in *PPP2R2A* KD cells. (**D**) Double-stranded DNA breaks in *PPP2R2A* KD OVCAR3 cells. Quantification of olive tail moment in OVCAR3 cells with or without CHK1 inhibition (1 µmol/L of LY2603618) for 2 h. (**E-I**) The extent of RS maker foci and staining density in CHK1i-treated *PPP2R2A* KD cells. The percentages of cells with positive γH2AX and pRPA2 S33 foci (≥ 5) (**E, F**) and the staining density of γH2AX and p-RPA2 S33 (**G, H**) in OVCAR3 cells with or without *PPP2R2A* KD using immunofluorescence assay. Cells were collected and fixed after treatment with the CHK1 inhibitor LY2603618 (1 μmol/L) for 2 h. Representative imaging of γH2AX and pRPA2 staining (**I**). Scale bar, 20 μm. Data in **B**, **D-H** are the mean ± SEM of three independent experiments. *n* = 3 in **D**-**F,** biological repeats; *n* = 300 in **G**, **H,** individual staining. Statistical significance was determined by one-way ANOVA, followed by Bonferroni post-hoc analysis for multiple comparisons. *, *P* < 0.05; **, *P* < 0.01; ***, *P* < 0.001; ****, *P* < 0.0001.

We proposed that reduced expression of PP2A B55α promotes oncogene-induced RS via upregulation of replication initiation, causing cells to be more dependent on CHK1 activity for survival. To test our hypothesis, we first assessed the RS induced by *PPP2R2A* KD by examining the levels of phosphorylated CHK1 at Ser345 (pCHK1), phosphorylated Replication Protein A2 (RPA2) at Ser 33 (pRPA2) and phosphorylated H2A.X Variant Histone (H2AX) at Ser139 (γH2AX), which serve as markers for RS and/or DNA double-strand breaks (DSBs). *PPP2R2A* stable KD alone resulted in the upregulation of pRPA2, γH2AX and pCHK1 (Ser 345) in three HGSOC cell lines compared to their own control cells (Figure 3A-B). This result suggests that *PPP2R2A* KD induces spontaneous RS and activates CHK1 without the challenge of exogenous DNA damage. Next, we determined whether CHK1 inhibition affects the RS in the cells with *PPP2R2A* KD. Treatment with a CHK1 inhibitor enhanced the expression of pRPA2, γH2AX and pCHK1, particularly in *PPP2R2A* KD HGSOC cells (Figure 3C). Of note, phosphorylated Checkpoint Kinase 2 (pCHK2) was also increased in the cells with CHK1 inhibitor treatment, but we did not see an obvious difference in cells with or without PPP2R2A KD. This may be caused by the time points since DNA damage response is a highly dynamic process. Additionally, utilizing a comet assay under neutral conditions, we observed a significant increase of DSBs in *PPP2R2A* KD OVCAR3 cells, compared to control cells without *PPP2R2A* KD (Figure 3D, Figure S6A). Treatment with CHK1 inhibitors resulted in a more pronounced increase of DSBs in *PPP2R2A* KD cells (Figure 3D, Figure S6A). Furthermore, we found a significant increase in the percentage of cells exhibiting positive staining for pRPA2 and γH2AX foci or pan-staining in *PPP2R2A* KD OVCAR3 and PEO4 cells using immunostaining (Figure 3E-I, Figure S6B-E). Pan-nuclear γH2AX can be used as a marker of widespread replication fork collapse during RS 47. We found a more substantial increase in pRPA2 and γH2AX foci (Figure 3E- F), as well as staining density (Figure 3G-H, Figure S6C-D), induced by CHK1 inhibition in OVCAR3 and PEO4 cells with *PPP2R2A* KD compared to control (Figure 3I, Figure S6E). A similar result was observed when we used a second shRNA to target *PPP2R2A* (Figure S6B-E). Additionally, in OV90 HGSOC cells with spontaneously low expression of B55α, CHK1 inhibition also led to increased expression of RS/DNA damage markers (Figure S6F). The staining intensity of pRPA2 and γH2AX was upregulated in OV90 cells following CHK1 inhibitor treatment (Figure S6G-I).

Additionally, we assessed cell proliferation alteration induced by CHK1 inhibition in cells with or without *PPP2R2A* KD using BrdU incorporation assay. The number of BrdU-positive cells was significantly lower in CHK1 inhibitor-treated OVCAR3 cells with *PPP2R2A* KD compared to CHK1 inhibitor-treated control OVCAR3 cells (Figure S7A-C). Collectively, these findings suggest that *PPP2R2A* KD heightens RS without the presence of external DNA damaging agents, leading to the activation of CHK1. Therefore, further CHK1 inhibition results in a more substantial increase in RS and cell proliferation defect in *PPP2R2A* KD HGSOC cells, compared to its control cells with intact PPP2R2A expression.

### *PPP2R2A* KD enhances replication initiation, a phenomenon that is further increased by CHK1 inhibition

**Figure 4:**
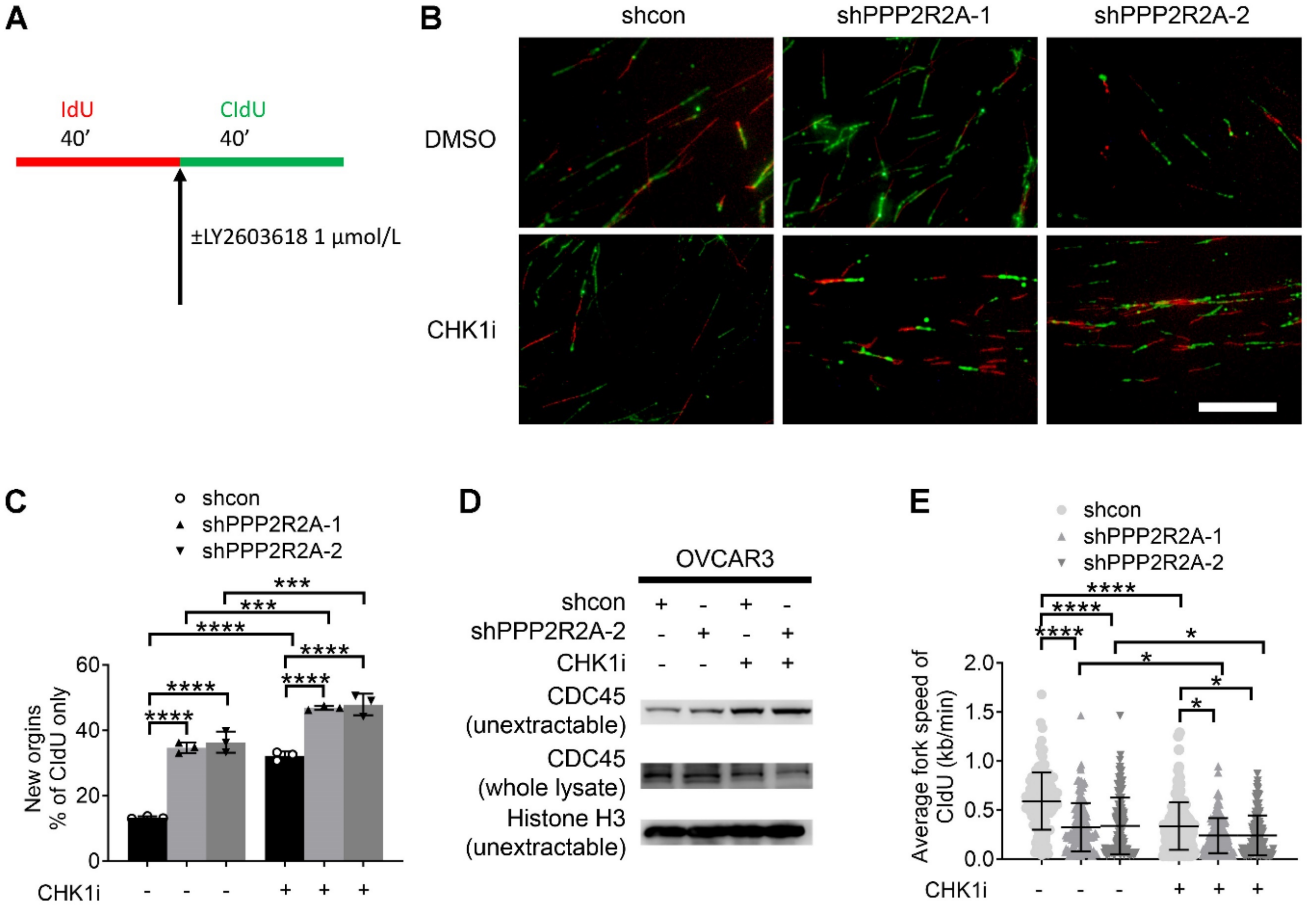
** CHK1 inhibition disrupts the replication fork dynamics, particularly in *PPP2R2A* KD OVCAR3 cells**. (**A**) A schematic diagram illustrating the labeling scheme: IdU is incorporated as the first analog for 40 min, followed by incorporation of CldU as the second analog plus CHK1 inhibitor treatment for 40 min. (**B**) Representative images of DNA fibers from OVCAR3 cells treated with DMSO or LY2603618 (1 μmol/L). Scale bar, 100 μm. (**C**) The extent of replication initiations in OVCAR3 cells treated with the CHK1 inhibitor LY2603618. *n* = 3, biological repeats. (**D**) Chromatin loading of CDC45 in *PPP2R2A* KD cells after 2 h of CHK1i (1 μmol/L) treatment. (**E**) Average fork speed in *PPP2R2A* KD cells treated with or without a CHK1 inhibitor compared to control cells. *n* = 300 in **E**, individual counting of each fiber from three biological repeats. Data in **C**, **E** are the mean ± SEM of three independent experiments. Statistical significance was determined by one-way ANOVA, followed by Bonferroni post hoc analysis for multiple comparisons. *, *P* < 0.05; ***, *P* < 0.001; ****, *P* < 0.0001.

Deregulated replication initiations are a cause of RS 48. To elucidate the mechanisms by which *PP2R2A* KD increases RS, we first accessed the rate of DNA synthesis during S phase progression using a DNA fiber assay, as outlined in the labeling scheme indicated in Figure 4A. Representative DNA fiber images are shown in Figure 4B. The percentage of new origin firing is significantly increased in *PPP2R2A* KD cells, which was significantly increased by CHK1i especially in PPP2R2A KD cells (Figure 4C). CDC45 (Cell Division Cycle 45) is a rate-limiting factor for replication initiation and is linked to oncogene-induced replication initiation 49, 50. Accordingly, *PPP2R2A* KD induces the enrichment of CDC45 in chromatin using two different shRNAs, as confirmed by western blot analysis following fractionation (Figure 4D). CHK1 inhibition further heightened the levels of non-extractable CDC45 (Figure 4D).

The chromatin loading of CDC45 results in excessive origin firing, subsequently leading to a reduction in elongation rate and the emergence of replication fork asymmetries. We observed a significant decrease in the elongation rate of CIdU (chlorodeoxyuridine) labeling in PPP2R2A KD cells, and CHK1 inhibition further exacerbates the reduction of fork speed in CIdU labeling (Figure 4E).

In summary, our data collectively indicate that *PPP2R2A* KD disrupts replication dynamics by elevating the degree of replication initiations and reducing replication fork speed. CHK1 inhibition further intensifies replication initiations, exacerbates the reduction in replication fork speed. The perturbation in replication fork dynamics is attributed to the RS triggered by *PPP2R2A* KD. These findings are consistent with the increased sensitivity to CHK1i in PPP2R2A KD cells, compared to control cells without PPP2R2A KD.

### Oncogene c-Myc is elevated in *PPP2R2A* KD HGSOC cells

**Figure 5:**
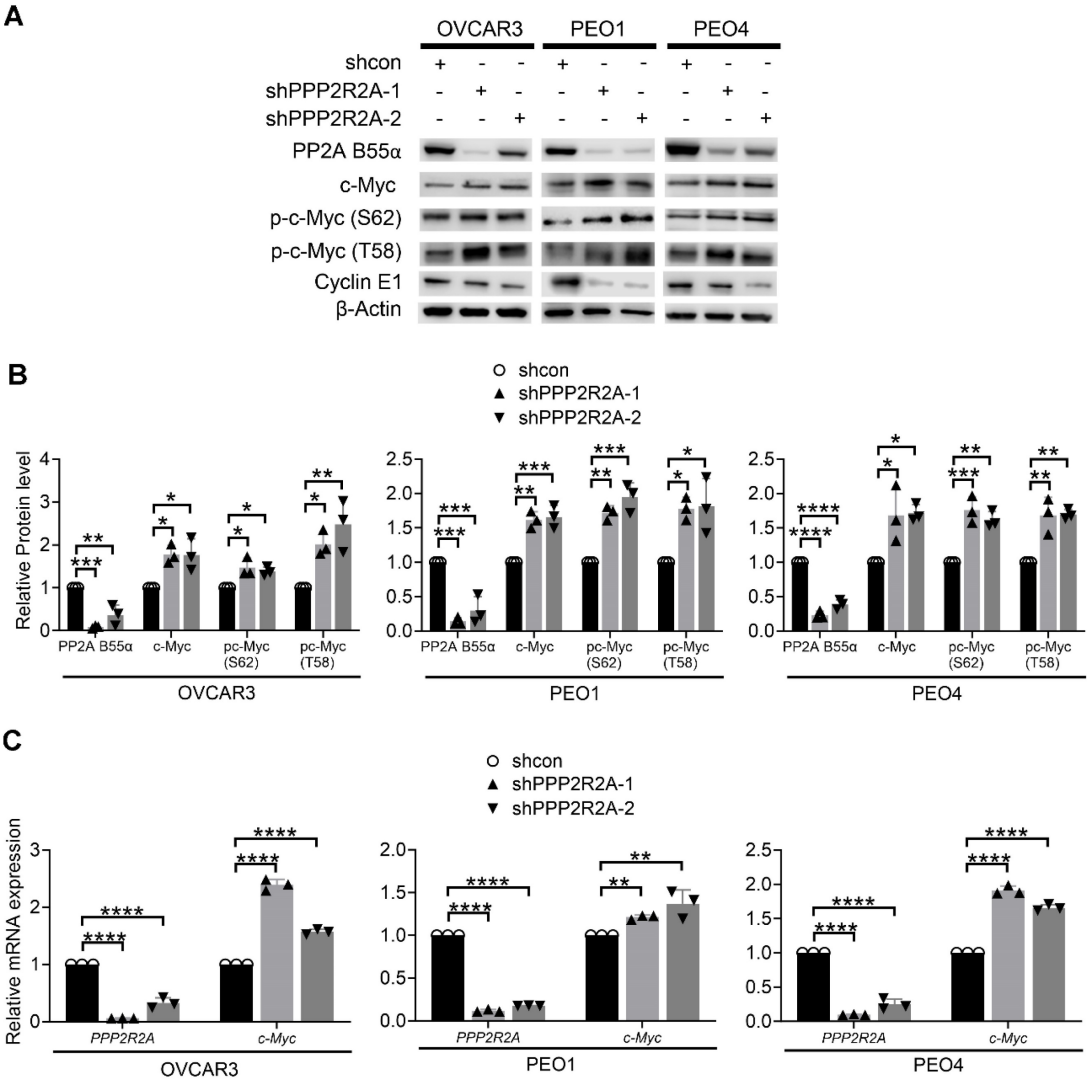
** Oncogene c-Myc is significantly increased in *PPP2R2A* KD HGSOC cells.** (**A**) Phosphorylated c-Myc and total c-Myc levels in *PPP2R2A* KD HGSOC cells as determined by immunoblot. (**B**) Densitometric quantitation of Western blot analysis of c-Myc expression in *PPP2R2A* KD cells. Statistical analysis of expression of proteins relative to β-Actin in HGSOC cells with or without *PPP2R2A* KD. (**C**) *c-Myc* mRNA levels in* PPP2R2A* KD cells*.* The *PPP2R2A* and *c-Myc* expression, as detected by qRT-PCR, are normalized to *GAPDH* in HGSOC cells. *n* = 3 in **B**, **C,** biological repeats. Statistical significance in **B**, and** C** was determined by one-way ANOVA, followed by Bonferroni post-hoc analysis for multiple comparisons. *, *P* < 0.05; **, *P* < 0.01; ***, *P* < 0.001; ****, *P* < 0.0001.

Next, we sought to elucidate how *PPP2R2A* KD induces spontaneous RS in HGSOC cells. The levels of c-Myc, a well-known oncogene associated with RS, are higher after the loss of PP2A B55α expression in NSCLC cells^39^. Thus, we next determined the involvement of c-Myc in *PPP2R2A* KD-induced RS in HGSOC cells. Firstly, we measured c-Myc protein expression in the cells with or without *PPP2R2A* KD. *PPP2R2A* KD by two different shRNAs resulted in higher c-Myc protein expression in the three HGSOC cell lines (Figure 5A-B). c-Myc stabilization requires phosphorylation of c-Myc at serine 62 (S62), which inhibits its ubiquitination-dependent degradation. Threonine 58 (T58) phosphorylation necessitates prior S62 phosphorylation, and a single T58 phosphorylation of c-Myc makes it a substrate for proteasome-mediated ubiquitination and degradation. This dually phosphorylated form of c-Myc associates with the higher transcriptional activation compared to mono phosphorylated form 51. We have demonstrated that in NSCLC, *PPP2R2A* KD induces the phosphorylation of c-Myc at both S62 and T58, without influencing the protein stability of c-Myc 39. Similar results were also observed in three HGSOC cell lines with *PPP2R2A* KD. The phosphorylation of c-Myc at both S62 and T58 was greater in *PPP2R2A* KD HGSOC cells compared to the control shRNA-treated cells (Figure 5A-B). The *PPP2R2A* KD had no impact on the protein stability of c-Myc, as detected by a cycloheximide block protein synthesis assay (Figure S8A-H). However, in the three HGSOC cell lines, *PPP2R2A* KD led to an increase in *MYC* mRNA (Figure 5C), which differs from what we observed in NSCLC in that no change in *MYC* mRNA expression were observed in *PPP2R2A*-KD NSCLC cells 39. Of note, in addition to c-Myc, Cyclin E overexpression is associated with RS 8. However, we found a decreased Cyclin E expression in the cells with *PPP2R2A* KD (Figure 5A); thus, Cyclin E may not be important for *PPP2R2A* deficiency-induced RS. Therefore, these data suggest that *PPP2R2A* KD leads to increased c-Myc expression via regulation of its mRNA expression, although the molecular mechanism controlling this remains unknown.

Given that PP2A B56α is reported to negatively regulate c-Myc phosphorylation and knockdown of *PPP2R5A*, the gene encoding PP2A B56α, resulted in the elevation of c-Myc protein 52, we next determined if *PPP2R2A* KD-induced c-Myc expression is associated with B56α. We found that *PPP2R2A* KD still resulted in the upregulated expression of c-Myc protein even in the cells with *PPP2R5A* stable KD (Figure S8I-J). Collectively, these results demonstrate that *PPP2R2A* depletion negatively regulates c-Myc protein and/or transcriptional expression, and this regulation is independent of c-Myc protein degradation and PP2A B56α levels.

Given that we found that PPP2R2A KD leads to increased c-Myc expression, we next also determined if there is an association between the expression of these two proteins among the cell lines we used in this study. We examined c-Myc expression in OVCAR3, PEO1, PEO4, and two cell lines with lower PPP2R2A expression (*i*.*e*., OV90 and CAOV3)**.** Unlike isogenic paired cell lines, such as OVCAR3, PEO1 and PEO4, parental cells versus their own PPP2R2A KD cell pair (Figure S5A) (namely, OV90 and CAOV3) did not exhibit absolutely elevated levels of c-Myc and RS markers, compared to other cell lines with relatively higher B55 α (OVCAR3, PEO1 and PEO4) (Figure S5A). This discrepancy is due to the complexity of the genetic backgrounds among different cell lines. Comparing protein expression association across limited cell lines with different genetic backgrounds can be challenging. PPP2R2A expression status is not the only factor that shows such a difference and the difference with other potential factors co-exist. In addition to c-Myc, other oncogenes could also be responsible for the increased RS and the sensitivity to CHKi. Despite this, we still observed that CHK1 inhibition suppressed the proliferation of OV90 and CAOV3 cells (Figure S5D-E) whereas CHK1 inhibition had no obvious impact on the parental cell lines with relatively higher PPP2R2A expression (Figure S5F-H). Furthermore, CHK1 inhibition increased RS markers in OV90 cells (Figure S6F-I). Of note, for some assays we only used OV90 because CAOV3 growth is extremely slow, which prevents its utility for extensive analysis. Thus, an extensive array of HGSOC cell lines is needed in future studies to further test the association between PPP2R2A/B55α and c-Myc expression.

### PPP2R2A KD-induced RS is dependent on c-Myc

Given that *PPP2R2A* KD upregulates c-Myc expression in HGSOC cells, we hypothesized that c-Myc activity contributes to PPP2R2A KD/deficiency-induced RS and the increased sensitivity to CHK1i treatment. Therefore, when c-Myc activity is inhibited, the RS and CHK1 sensitivity induced by PPP2R2A KD/deficiency are reduced. Oncogenes, including c-Myc, can cause RS by upregulating replication initiation, which can lead to dNTP pool deficiency and conflicts with transcription. If c-Myc is required for PPP2R2A KD-induced replication initiation, then inhibiting c-Myc should reduce PPP2R2A KD-induced replication initiation, subsequent RS, and sensitivity to CHK1i treatment.

**Figure 6:**
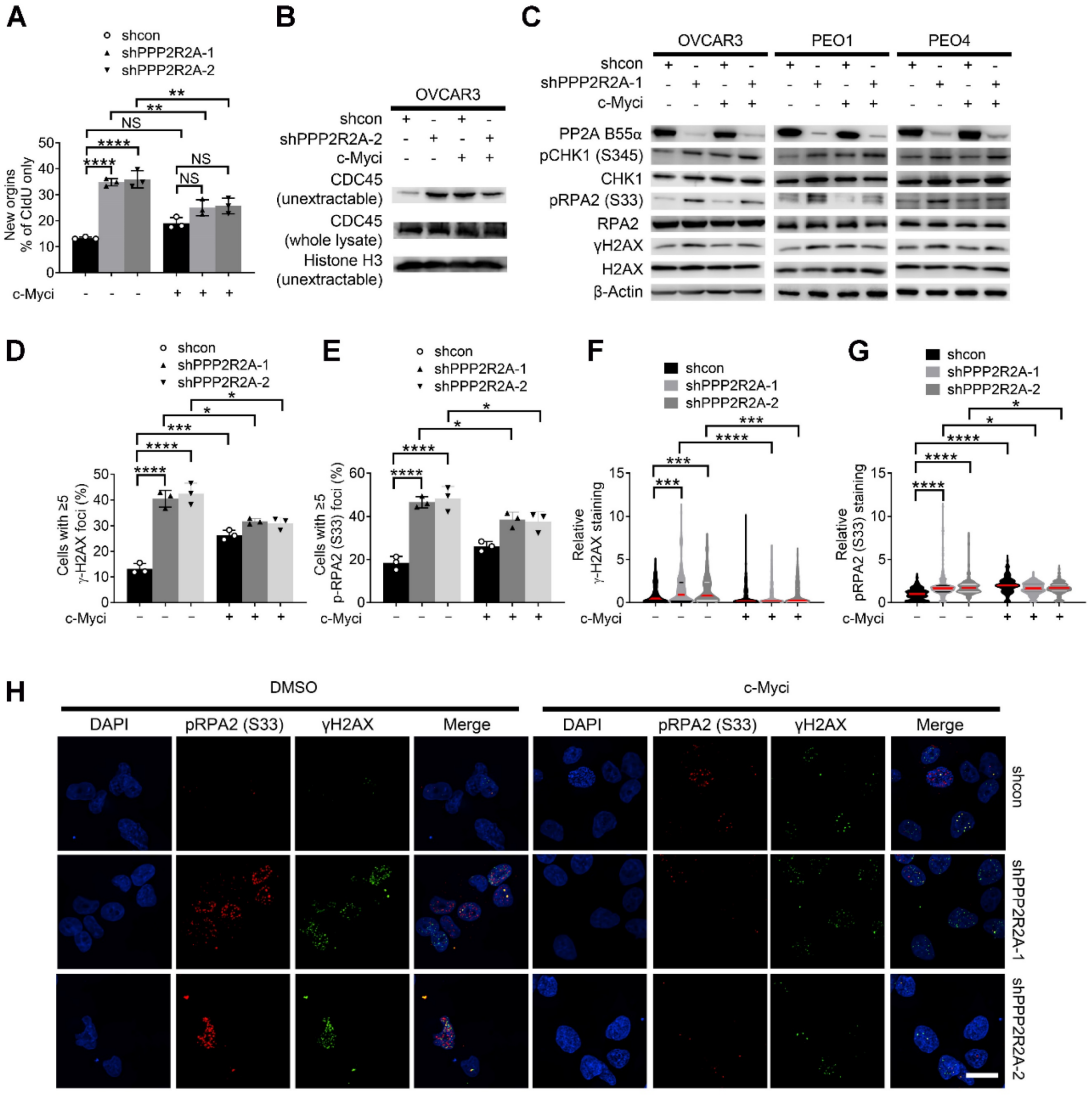
** The inhibition of c-Myc abolishes *PPP2R2A* KD-triggered RS in OVCAR3 cells.** (**A**) Replication initiations of c-Myc inhibitor-treated *PPP2R2A* KD cells. Statistical significance was determined by one-way ANOVA, followed by Bonferroni post hoc analysis for multiple comparisons. *n* = 3, biological repeats; **, *P* < 0.01; ****, *P* < 0.0001. (**B**) CDC45 chromatin loading in c-Myc inhibitor-treated OVCAR3 cells (10058-F4; 20 μmol/L) for 2 h with or without PPP2R2A KD. (**C**) RS marker expression in *PPP2R2A* KD HGSOC cells treated with a c-Myc inhibitor (10058-F4; 20 μmol/L) for 2 h). (**D-H**) RS maker foci and staining density in PPP2R2A KD cells treated with a c-Myc inhibitor (10058-F4; 20 μmol/L) for 2 h. The percentages of cells with positive γH2AX and pRPA2 S33 foci (≥5) (**D-E**) and the staining density of γH2AX and p-RPA2 S33 (**F-G**) in OVCAR3 cells with or without *PPP2R2A* KD as assessed by immunofluorescence assays. Cells were collected and fixed after treatment with the c-Myc inhibitor. Data in **D-G** are the mean ± SEM of three independent experiments. *n* = 3 in **D-E**, biological repeats; *n* = 300 in **F-G**, individual staining. Statistical significance was determined by one-way ANOVA, followed by Bonferroni post-hoc analysis for multiple comparisons. *, *P* < 0.05; ***, *P* < 0.001; ****, *P* < 0.0001. Representative images of γH2AX and pRPA2 staining are shown in (H). Scale bar, 20 μm.

To test this hypothesis, we initially evaluated the impact of c-Myc pharmacological inhibition on *PPP2R2A* KD-induced RS and increased sensitivity to CHK1 inhibition. Firstly, we found that the c-Myc inhibitor 10058-F4 abolished *PPP2R2A* KD-induced replication initiation, as demonstrated by a DNA fiber assay (Figure 6A, Figure S9A). Representative DNA fiber images are show in Figure S9B. However, c-Myc inhibition had no effect on the CIdU fork speed because *PPP2R2A* KD still led to a slow average fork speed regardless of the c-Myc inhibition (Figure S9C). This could be caused by the dynamics and transient nature of replication fork stalling; the replication initiation and fork speed might not occur simultaneously and the regulations on the replication initiation and replication elongation can be dissociated. Secondly, we found that c-Myc inhibition abrogated *PPP2R2A* KD-induced increase in non-extractable CDC45 in OVCAR3 cells (Figure 6B), which is consistent with the decreased replication initiation observed by DNA fiber assay. To validate this result from the DNA fiber assay, we further assessed whether c-Myc inhibition affects *PPP2R2A* KD-induced expression of pRPA2 and γH2AX. *PPP2R2A* depletion-induced pRPA2 and γH2AX expression were reduced in the cells with c-Myc inhibition (Figure 6C).

Phosphorylation of c-Myc-S62 is crucial for both c-Myc stability and transcriptional activation 51. To validate our results observed with c-Myc inhibition, we next examined the influence of stable overexpression of c-Myc WT and a c-Myc S62A mutant on RS triggered by *PPP2R2A* KD. The levels of p-PRAP2 and γH2AX induced by *PPP2R2A* KD was lower in the cells expressing the c-Myc S62A mutant compared to the cells expressing c-Myc-WT. Therefore, genetic inactivation of c-Myc abolished the *PPP2R2A* KD-induced expression of pRPA2 and γH2AX (Figure S9D).

To support this result, we further determined the impact of c-Myc inhibition on *PPP2R2A* KD-induced RS protein foci using immunofluorescence staining (Figure 6D-H). We found that c-Myc inhibition reduced the *PPP2R2A* KD-induced pRPA2 and γH2AX foci formation (Figure 6D-E), and the intensity of these protein staining (Figure 6F-G). In support of the involvement of c-Myc, its inhibition also led to decreased expression of RS markers in OV90 cells (Figure S10A). Furthermore, c-Myc inhibition reduced the intensity of pRPA2 and γH2AX in OV90 cells (Figure S10B-D).

To further determine the involvement of c-Myc activity, we next examined the impact of c-Myc inhibition on the cell proliferation of OVCAR3 cells with or without *PPP2R2A* KD using FACS analysis to detect the BrdU labeling (Figure S11). The decreased BrdU labeling in *PPP2R2A* KD cells was restored in the cells treated with the c-Myc inhibitor (Figure S11A-C).

### *PPP2R2A* KD-induced sensitivity to CHK1 inhibition is dependent on c-Myc

**Figure 7:**
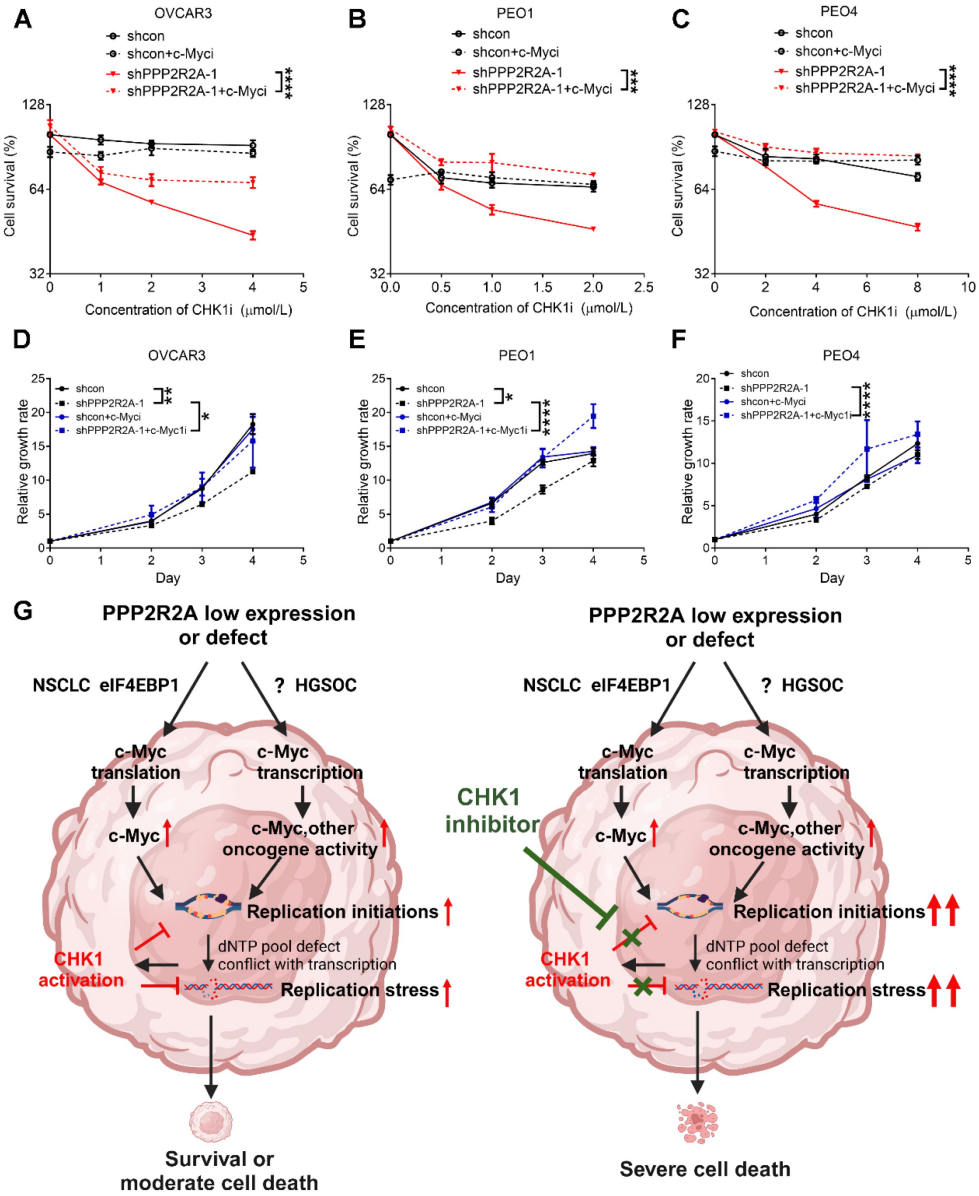
** The inhibition of c-Myc mitigates *PPP2R2A* KD-induced sensitivity to CHK1 inhibition.** (**A-C**) c-Myc inhibitor treatment decreases the PPP2R2A KD-triggered sensitivity to CHK1 inhibition in OVCAR3 (**A**), PEO1 (**B**), and PEO4 (**C**) cells. Cell survival was assessed in the indicated cells with or without PPP2R2A deficiency. These cells were treated with either a c-Myc inhibitor (10058-F4; 20 μmol/L) or a CHK1 inhibitor for 48 h. (**D-F**) Relative growth of the indicated cell lines with or without *PPP2R2A* deficiency and treated with a c-Myc inhibitor (10058-F4; 20 μmol/L) for 48 h. *n* = 3, biological repeats; *, *P* < 0.05, **, *P* < 0.01, ***, *P* < 0.001, ****, *P* < 0.0001, two-way ANOVA, followed by Bonferroni post-hoc analysis for multiple comparisons was used to determine statistical significance in (**A-F**). (**G**) A schematic diagram illustrating the proposed working model regarding the increased sensitivity to CHK1 inhibition in the HGSOC cells with PPP2R2A KD or deficiency. This image was generated using BioRender (https://biorender.com/).

Finally, we investigated whether c-Myc is crucial for *PPP2R2A* depletion-induced sensitivity to CHK1 inhibition. We found that c-Myc inhibition reduced the *PPP2R2A* KD-induced sensitivity to CHK1 inhibitors in three HGSOC cells (Figure 7A-C, Figure S12A-C). In support of the result that c-Myc is important for the RS induced by PPP2R2A deficiency, treatment with a c-Myc inhibitor increased the cellular growth of OVCAR3 (Figure 7D, Figure S12D), PEO1 (Figure 7E, Figure S12E) and PEO4 (Figure 7F, Figure S12F) cells with *PPP2R2A* KD. Additionally, c-Myc inhibition also promoted the proliferation of OV90 and CAOV3 cells (Figure S12G-H) although such inhibition had no obvious impact on HGSOC cells with intact *PPP2R2A* expression (Figure S12I-K).

Taken together, these findings indicate that c-Myc is involved in *PPP2R2A* KD/low expression-induced RS. The c-Myc activity is required for CHK1 inhibition-induced RS and interruption of cell growth, especially in HGSOC cells with *PPP2R2A* KD/B55 α low expression.

## Discussion

The function of PP2A is highly context-dependent, varying across different cell types. PP2A deficiency can result in either drug sensitivity or resistance, among different type of cancer. Although our studies have shown that PPP2R2A deficiency affects the sensitivity to CHK1 inhibitors in NSCLC, its impact on HGSOC remains unknown. Notably, clinical trials have been conducted in HGSOC, and a subtype of patients have shown promising responses to CHK1 inhibitors 22. However, there is a lack of biomarker studies in this context. Therefore, it is crucial to determine the impact of PPP2R2A deficiency in ovarian cancer. Understanding whether PPP2R2A deficiency affects sensitivity to CHK1 inhibitors in HGSOC is important.

Most patients with HGSOC experience a relapse due to tumor resistance despite initial responses to cytoreductive surgery, platinum-based chemotherapy and PARP inhibitor-based target therapy. *PPP2R2A*/PP2A B55α is frequently deleted or under-expressed in various human cancers, including ovarian cancer 31. Additionally, low expression of *PPP2R2A* is linked to poor prognosis in multiple cancer types, including HGSOC. To explore potential therapies for HGSOC with *PPP2R2A* deficiency, we assessed the impact of *PPP2R2A* KD on CHK1 inhibitor sensitivity in HGSOC and found it increases such sensitivity, including in PARP inhibitor-resistant HGSOC cell lines, through upregulation of c-Myc-induced oncogenic RS. This implies a potential therapeutic target for HGSOC, particularly those with *PPP2R2A* low expression/deficiency, and suggests such deficiency as a potential biomarker for guiding CHK1 inhibitor use in treating HGSOC.

During the cell cycle, when cells face exogenous DNA damage, they undergo lesions that require repair before entering mitosis to maintain genome integrity in daughter cells. Cells have developed a complex DDR mechanism, involving direct DNA repair, cell cycle checkpoints, transcriptional regulation and apoptosis. Initially, it was thought that p53 is important for the G1/S checkpoint and its loss of expression leads to disruption of this cellular checkpoint, leaving cells reliant on cell cycle G2/M arrest for DNA repair when the cells are challenged with DNA damaging agents 53-55. CHK1 phosphorylates and inhibits its substrates, the phosphatases CDC25C and CDC25A, leading to arrest at the G2/M checkpoint 55. Therefore, CHK1 inhibitors could have anti-tumor properties in p53-deficient cancer cells if used in combination with standard chemotherapy and radiation therapy as these agents induced DNA damage and such cells are highly reliant on repair mechanisms during the G2/M phase and intra S phase for their survival 55.

The nearly universal loss of normal p53 regulation in HGSOCs could lead to the interruption of the G1/S checkpoint, rendering the tumor cells reliant on CHK1-mediated G2/M arrest for DNA damage repair when the cancer cells are challenged by exogenous DNA damaging agents. However, recent studies suggest that p53 is also important for the G2/M phase checkpoint 56. CHK1 inhibitors also have an antitumor activity as a monotherapy, even without combination with the exogenous DNA damage agents, in cells with p53 proficiency 55, suggesting the existence of unrelated mechanisms for cell cycle arrest that contribute to its antitumor activity. Indeed, accumulated evidence to date suggest that CHK1 signaling is important for the cell's growth even in the condition without challenge by exogenous DNA damage 55. Our findings here suggest that CHK1 inhibition alone is sufficient to target *PPP2R2A*-KD/low expression HGSOC cells, due to its impact on the replication initiation and subsequent escalation in RS. Therefore, use of a CHK1 inhibitor as a monotherapy target *PPP2R2A*-low expressing/deficient HGSOC cells by upregulation of replication initiation-associated RS. However, we cannot exclude other potential mechanisms that contribute to the *PPP2R2A* deficiency-induced CHKi sensitivity. For example, HR activity is impaired in *PPP2R2A*-deficient cells 31. ATR/CHK1 inhibition has been reported to specifically target HR-deficient cells 57 and increases the toxicity of PARP inhibition by preventing HR protein Rad51-mediated foci formation in wild-type BRCA-expressing HGSOC 58. While HR activity impairment is observed in *PPP2R2A*-deficient lung cancer cells 31, it may not be a major factor in CHK1 inhibitor sensitivity of HGSOC , as HR status did not influence the *PPP2R2A* KD-mediated CHK1 inhibitor sensitivity in our study here.

PP2A has diverse functions, including the negative regulation of numerous oncogenic signaling. Oncogenic activation causes endogenous RS that could be lethal to the cells. CHK1 signaling suppresses RS to less toxic levels. Given the role of PP2A in negatively regulating multiple oncogenic pathways 24, 38, 59, *PPP2R2A* deficiency may lead to oncogene activation and RS, rendering these cells sensitive to CHK1 inhibition. It has been demonstrated that PP2A B55α regulates the activity of key oncogene proteins, including AKT Serine/Threonine Kinase (Akt), Ras/ Mitogen-Activated Protein Kinase (Mapk), SRC Proto-Oncogene, Non-Receptor Tyrosine Kinase (Src), Mechanistic Target Of Rapamycin Kinase (mTOR) and Wingless-Type MMTV Integration Site Family (Wnt)/Cadherin-Associated Protein, Beta 1 (b-Catenin) 24, 59, 60. Our previous study suggests that PP2A B55α regulates c-Myc expression in NSCLC 39 and cMyc activity is required for *PPP2R2A* deficiency-induced replication initiation/RS and *PPP2R2A* low expression/deficiency-induced CHK1 inhibitor sensitivity in NSCLC 39. A similar pattern was observed in HGSOC where PPP2R2A low expression increases the sensitivity to CHK1i treatment (Figure 7G). Thus, despite the complexity of *PPP2R2A* deficiency in the regulation of oncogenic signaling and cancer therapy in the different types of cancer, its impact on the increased sensitivity to CHK1 inhibition in NSCLC and HGSOC appears to be similar.

Nevertheless, we recognize the difference in the mechanisms by which PPP2R2A KD induces c-Myc expression in NSCLC and HGSOC cells. Although this study is not intended to compare the regulation of c-Myc between NSCLC and HGSOC cell lines, we observed that the upregulation of c-Myc in PPP2R2A-deficient cells occurs through an eIF4EBP1-associated translation mechanism in NSCLC 39, whereas in HGSOC, a transcriptional regulatory mechanism is involved (Figure 5). Additionally, although c-Myc expression is increased in different HGSOC cell lines with *PPP2R2A* KD and c-Myc is required for *PPP2R2A* deficiency-induced RS, the mild increase in c-Myc expression in PPP2R2A KD cells in HGSOC suggests that other oncogenes, which are upregulated in such cells, may also play a significant role in the sensitivity to CHK1i treatment that is induced by PPP2R2A KD (Figure 7G). Moreover, the status of p53 could also be a factor affecting CHK1i sensitivity. The p53 mutation rate in NSCLC is approximately 46% in adenocarcinoma and 90% in squamous cell carcinoma 61 whereas HGSOC universally display p53 mutation 62 . Thus, further research is needed to investigate how PPP2R2A KD leads to increased c-Myc transcription and the involvement of other potential factors. Additionally, although it has been reported that PPP2R2A KD leads to increased sensitivity to PARP inhibition due to its impact on HR 31, we found that PPP2R2A KD has no impact on PARP inhibition in HGSOC cells.

It is noteworthy that while increased replication initiation is involved in the increased sensitivity to CHK1i in the cells with *PPP2R2A* KD or deficiency (Figure 7G), we cannot exclude other potential mechanisms that could also be important. For instance, the roles of CHK1 in replication fork stability, dNTP pool maintenance and cell cycle checkpoints could also be important to the increased RS and cell proliferation defect due to tress in the CHK1 inhibitor treated cells with PPP2R2A deficiency. Additionally, we cannot exclude the involvement of senescence as G2 cell cycle arrest can cause senescence 63 and the checkpoint kinase CHK1 controls cyclin D-CDK activity during G2 arrest. CHK1 depletion promotes senescence as well 64.

Oncogenes trigger RS by causing aberrant origin firing/replication initiation, collisions between replication and transcription, impaired nucleotide metabolism and the elevated levels of reactive oxygen species 48. The precise mechanism by which oncogene stress enhances RS through the regulation of replication initiation is not fully understood. However, there is a suggestion that it is associated with Cyclin Dependent Kinase 2 (CDK2)-mediated deregulation of replication initiation/firing, resulting in the subsequent depletion of the dNTP pool and the formation of single-stranded DNA (ssDNA) and DSBs 65, 66. This process may also involve an elevated occurrence of collisions induced by active replication forks or interference between replication and transcription machineries. Specifically, c-Myc induces RS through the deregulation of replication initiation 67. It is most likely that c-Myc activity increases in PPP2R2A KD/deficient cells, leading to the upregulation of replication initiation and subsequent RS. Given that c-Myc is required for PPP2R2A KD-induced replication initiation, c-Myc inhibition should reduce PPP2R2A KD-induced increases in replication initiation and subsequent replication levels and the sensitivity to CHK1i treatment. In support of this hypothesis, *PPP2R2A* depletion increases replication initiation and CHK1 inhibition further elevates the replication initiation, with all these regulations dependent on c-Myc activity. Therefore, c-My activity contributes to PPP2R2A KD/deficiency-induced RS and the increased sensitivity to CHK1i treatment via replication initiation dysregulation.

A striking finding in our study is that *PPP2R2A* KD increases the sensitivity to CHK1 inhibition in PARP inhibitor-resistant cells. Even though several PARP inhibitors have been approved for advanced ovarian, breast and pancreatic cancer, there is still more than 40% of patients with BRCA-mutant tumors that show no initial response to PARPi-based therapy or their cancers develop resistance following treatment 68, 69. Thus, PARPi resistance appears to be nearly inevitable 4 and such drug resistance remains a challenge. Several major mechanisms contributing to PARPi resistance have been identified, including the restoration of BRCA1/2 functions and HR by reversion mutations and epigenetic modification, restoration of PARPylation and fork stability, upregulation of drug efflux pumps and pharmacological alterations, suggesting potential strategies to overcome PARPi resistance 4. Our study suggests that PARPi-resistant HGSOC cells that are due to BRCA2 mutation reversion 70 can be targeted by CHK1 inhibition, especially in *PPP2R2A* KD cells. In addition, CHK1i toxicity is independent of the HR status. Our result is supported by a Phase 2 study showing that CHK1 inhibitor use displays a notable anti-tumor activity as a monotherapy in patients with a subtype of recurrent HGSOC with wild-type BRCA. Most patients in this study received intensive drug treatment and developed drug resistance 22. In addition, in a Phase 1 study the CHK1 inhibitor prexasertib demonstrated clinical activity, particularly in combination with the PARPi olaparib, even in patients with PARPi-resistant HGSOC 71. Additionally, prexasertib has exhibited durable single-agent activity in a subset of patients with recurrent HGSOC, irrespective of clinical characteristics, BRCA status or prior therapies, including PARPi, as shown in a recent Phase 2 study 72. Therefore, CHK1 inhibition could likely target a subtype of HGSOC that develop drug resistance, especially for PARPi-resistant cells with *PPP2R2A* KD. Of note, in addition to PPP2R2A, p53 may be a potential impact factor for CHK1 inhibitor induced suppression of cell growth 55. Although a p53-independent mechanism is involved in the *PPP2R2A* deficiency-induced RS/DNA damage and replication initiation regulation, p53 can still be a contributing factor to synergy give its important role in apoptosis 73. Of course, other identified genetic changes could also contribute to these differences. Thus, the status of *PPP2R2A* expression, along with other key factors affecting CHK1i activity, should be considered for guiding the decision-making process in the clinical trials of CHKi use.

The current study of *PPP2R2A* deficiency in the context of cancer therapy, particularly in relation to DDR inhibitors, reveals a promising avenue for targeted treatment. *PPP2R2A* deficiency has been identified as a critical factor influencing the response of cancer cells to PAPR inhibition and CHK1 inhibition in preclinical models 31, 39. It will be very interesting to study the impact of *PPP2R2A* deficiency/low expression on other different DDR inhibitors. Additionally, PP2A activity is modulated by several endogenous inhibitors 24. It has been demonstrated that the endogenous inhibitor of PP2A is frequently over-activated in human tumors so testing the sensitivity of such tumors to DDR inhibitors, including CHK1i's, will be of future interest.

In summary, in various types of cancer cell lines, including NSCLC and HGSOC cells, *PPP2R2A*-low expressing/deficient cells exhibit a heightened sensitivity to CHK1i treatment, though with a different means of control of c-Myc expression. More importantly, the implications of *PPP2R2A* deficiency extend beyond a specific cancer type, suggesting a potential broad applicability of DDR inhibitors in treating diverse malignancies with this genetic alteration. These findings underscore the significance of understanding the molecular mechanisms associated with *PPP2R2A* deficiency-induced RS, offering a foundation for the development of targeted therapies that exploit vulnerabilities in *PPP2R2A*-deficient cells. As our understanding progresses, this knowledge may contribute to refining precision medicine approaches in cancer therapy, especially in overcoming challenges associated with PARPi resistance in HGSOC. Further investigation into the antitumor activity of CHK1i's in tumors with *PPP2R2A* deficiency is warranted and holds significant implications for clinical trials involving such inhibitors.

## Supplementary Material

Supplementary figures.

## Abbreviations

PPP2R2A: Protein Phosphatase 2 Regulatory Subunit Balpha
CHK1: Checkpoint kinase 1
HGSOC: High-grade serous ovarian cancer
PARP: Poly (ADP-ribose) polymerase
RS: replication stress
PP2A: serine/threonine protein phosphatase 2
NSCLC: non-small cell lung cancer
HR: homologous recombination
EOC: Epithelial ovarian cancer
KRAS: KRAS Proto-Oncogene, GTPase
BRAF: B-Raf Proto-Oncogene, Serine/Threonine Kinase
ATR: Ataxia-telangiectasia-mutated-and-Rad3-related kinase
DDR: DNA damage response
c-Myc: MYC Proto-Oncogene, BHLH Transcription Factor
Ras: KRAS Proto-Oncogene, GTPase
PPP2R2B: Protein Phosphatase 2 Regulatory Subunit Bbeta
BRCA2: Breast And Ovarian Cancer Susceptibility Protein 2
KD: knockdown
LOH: loss of heterozygosity
RPA2: Replication Protein A2
H2AX: H2A.X Variant Histone
CHK2: Checkpoint kinase 2
DSBs: DNA double-strand breaks
CDC45: Cell Division Cycle 45
PPP2R5A: Protein Phosphatase 2 Regulatory Subunit B'Alpha
BrdU: 5-Bromo-2-deoxyuridine
IdU: iododeoxyuridine
CIdU: chlorodeoxyuridine
CDC25C: Cell Division Cycle 25C
CDC25A: Cell Division Cycle 25A
Akt: AKT Serine/Threonine Kinase
Mapk: Mitogen-Activated Protein Kinase
Src: SRC Proto-Oncogene, Non-Receptor Tyrosine Kinase
mTOR: Mechanistic Target of Rapamycin Kinase
Wnt: Wingless-Type MMTV Integration Site Family
Catenin: Catenin (Cadherin-Associated Protein), Beta 1
CDK2: Cyclin Dependent Kinase 2
