# Supplementary material for: Low PPP2R2A expression promotes sensitivity to CHK1 inhibition in high-grade serous ovarian cancer
Source: Theranostics. 2024 Nov 4;14(19):7450–69. doi: 10.7150/thno.96879 (PMC11626944; doi:10.7150/thno.96879)
Supplement: Supplementary file 1 — Supplementary figures. [file thnov14p7450s1.pdf]

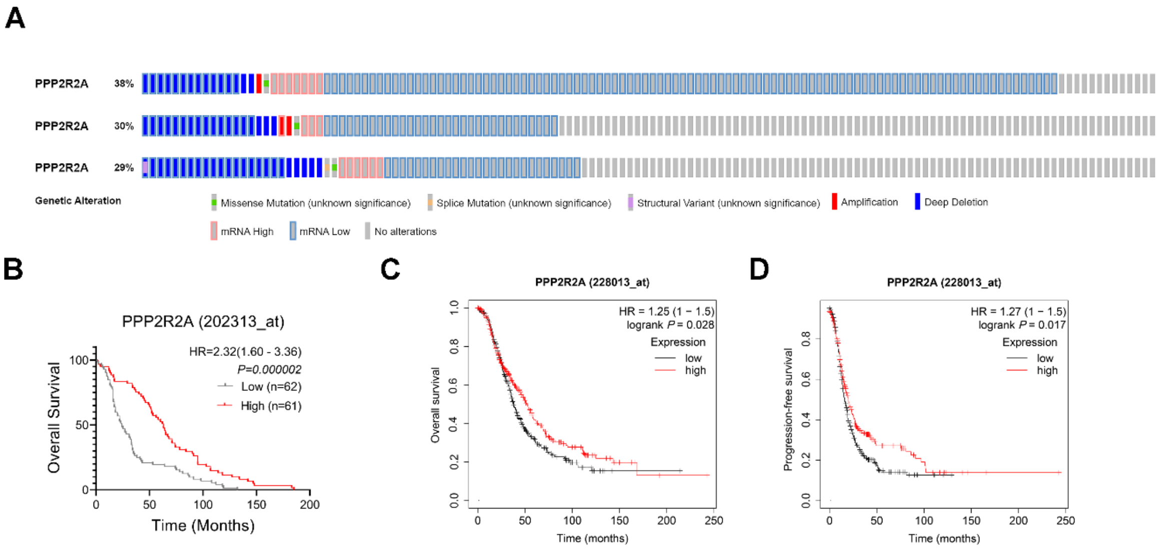


**Figure S1. *PPP2R2A* is downregulated in ovarian cancer and patient with ovarian cancer with low expression of *PPP2R2A* have a poor prognosis.** (**A**) Analysis of three TCGA datasets from cBioPortal indicates that *PPP2R2A* is frequently downregulated or deeply deleted in ovarian cancer. These datasets, listed from top to bottom, encompass Ovarian Serous Cystadenocarcinoma (TCGA, Nature 2011) with $n$ = 489, Ovarian Serous Cystadenocarcinoma (TCGA, Firehose Legacy) with $n$ = 617, and Ovarian Serous Cystadenocarcinoma (TCGA, PanCancer Atlas) with $n$ = 585. Kaplan-Meier survival analyses of ovarian cancer data indicate that patients with high PPP2R2A expression significantly improved overall survival (**B-C**) or progression-free survival (**D**). The *P*-value of **B**, obtained from PrognoScan, was determined using the Gehan-Breslow-Wilcoxon test, while the *P*-values of **C-D**, sourced from Kmplot, were calculated using the Log-rank test.

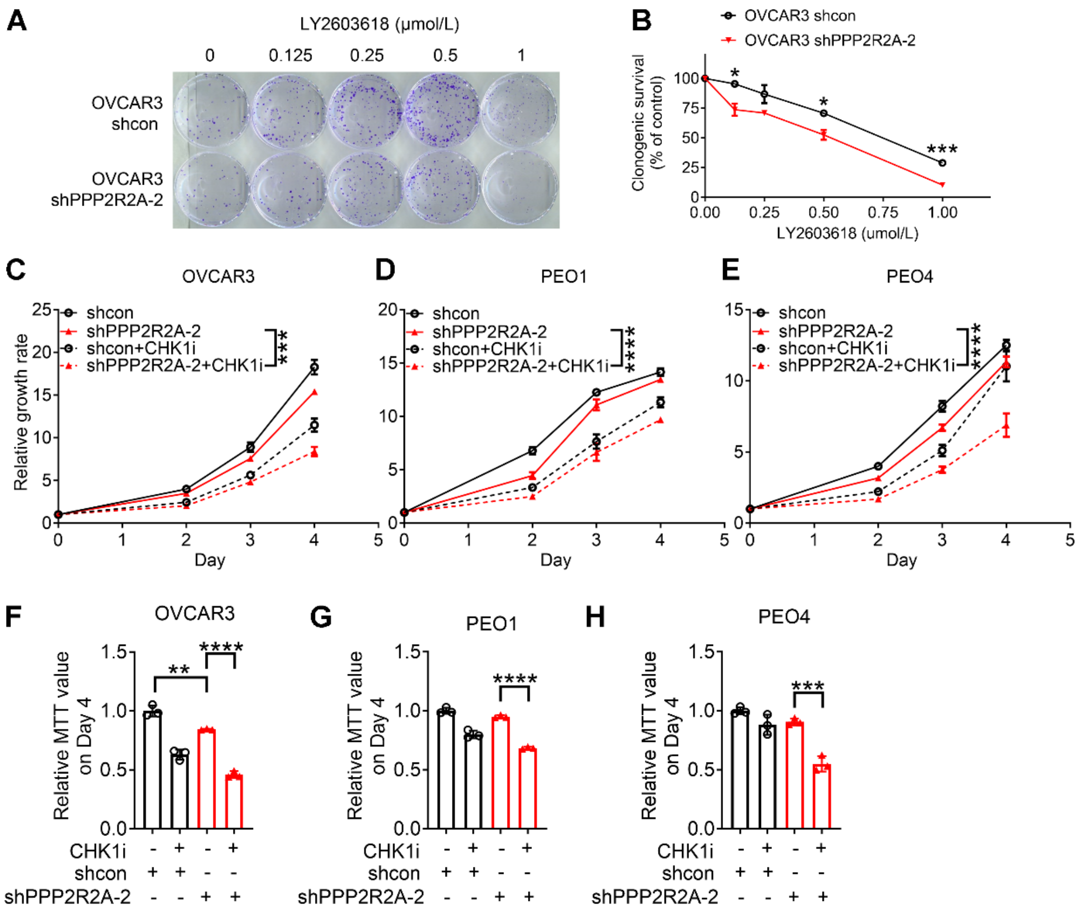


**Figure S2**. **Sensitivity to CHK1 inhibitor treatment in *PPP2R2A* knockdown ovarian cancer lines is confirmed using a second shRNA**. (**A-B**) Clonogenic assays for the CHK1 inhibitor treatment in the OVCAR3 cells with or without *PPP2R2A* knockdown (KD). Representative figures are shown in (**A**) and statistical analysis results are shown in (**B**). OVCAR3 cells were treated with 1 μmol/L of the CHK1 inhibitor LY2603618 for 24 h. Following this treatment, the cells were incubated in fresh medium for an additional 9 days. n=3, biological repeats (**B**). (**C-H**) CHK1i is more effective in the cells with *PPP2R2A* KD. Growth curves of OVCAR3 (**C**), PEO1 (**D**) and PEO4 (**E**) after *PPP2R2A* KD and CHK1 inhibition (1μmol/L for OVCAR3 and PEO4, 0.5 μmol/L for PEO1). Relative MTT values on day 4 for each cell lines are shown in (**F-H**). n=3, biological repeats (**C-H**). *, $P < 0.05$, **, $P < 0.01$, ***, $P < 0.001$, ****, $P < 0.0001$, two-way ANOVA, followed by Bonferroni post hoc analysis for multiple comparisons was used to determine statistical significance in (**C-E**); Statistical significance in (**B**, **F-H**) was determined by one-way ANOVA, followed by Bonferroni post hoc analysis for multiple comparisons.

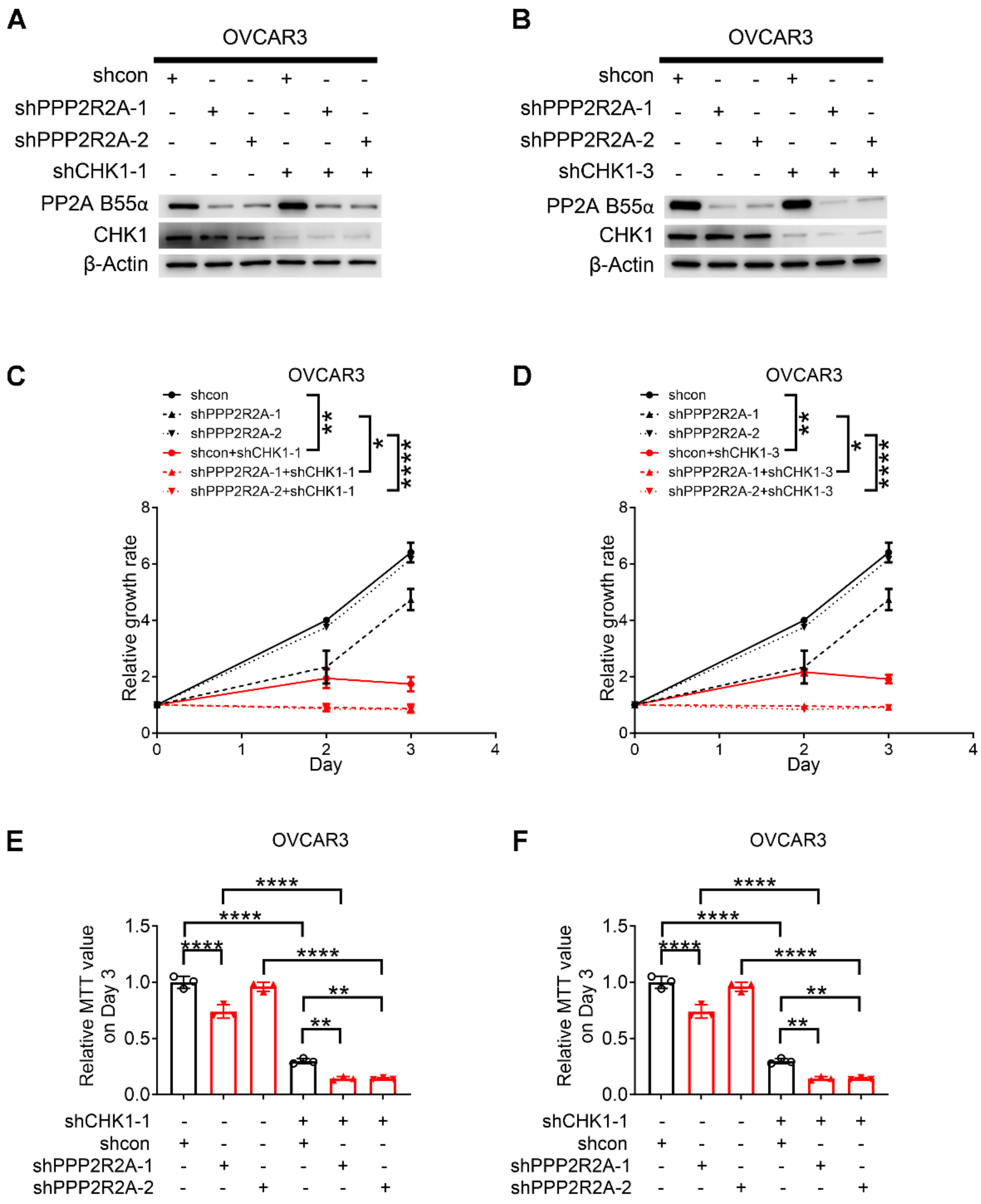


**Figure S3**. **CHK1 KD sensitizes OVCAR3 cells with *PPP2R2A* KD**. (**A**, **B**) Immunoblots demonstrate that CHK1 KD increases the γH2AX in OVCAR3 cells using two shRNAs targeting CHK1. (**C**, **D**) Knockdown of *CHK1* decreases the proliferation of OVCAR3 cells, particularly in those with *PPP2R2A* KD. $n$ = 3, biological repeats. *, $P < 0.05$, **, $P < 0.01$, ****, $P < 0.0001$, two-way ANOVA, followed by Bonferroni post-hoc analysis for multiple comparisons was used to determine statistical significance. (**E, F**) Relative MTT values on day 3 for each cell lines are shown in (**E-F**). $n$ = 3, biological repeats. **, $P < 0.01$, ****, $P < 0.0001$, one-way ANOVA,

followed by Bonferroni post-hoc analysis for multiple comparisons was used to determine statistical significance in **E**, **F**.

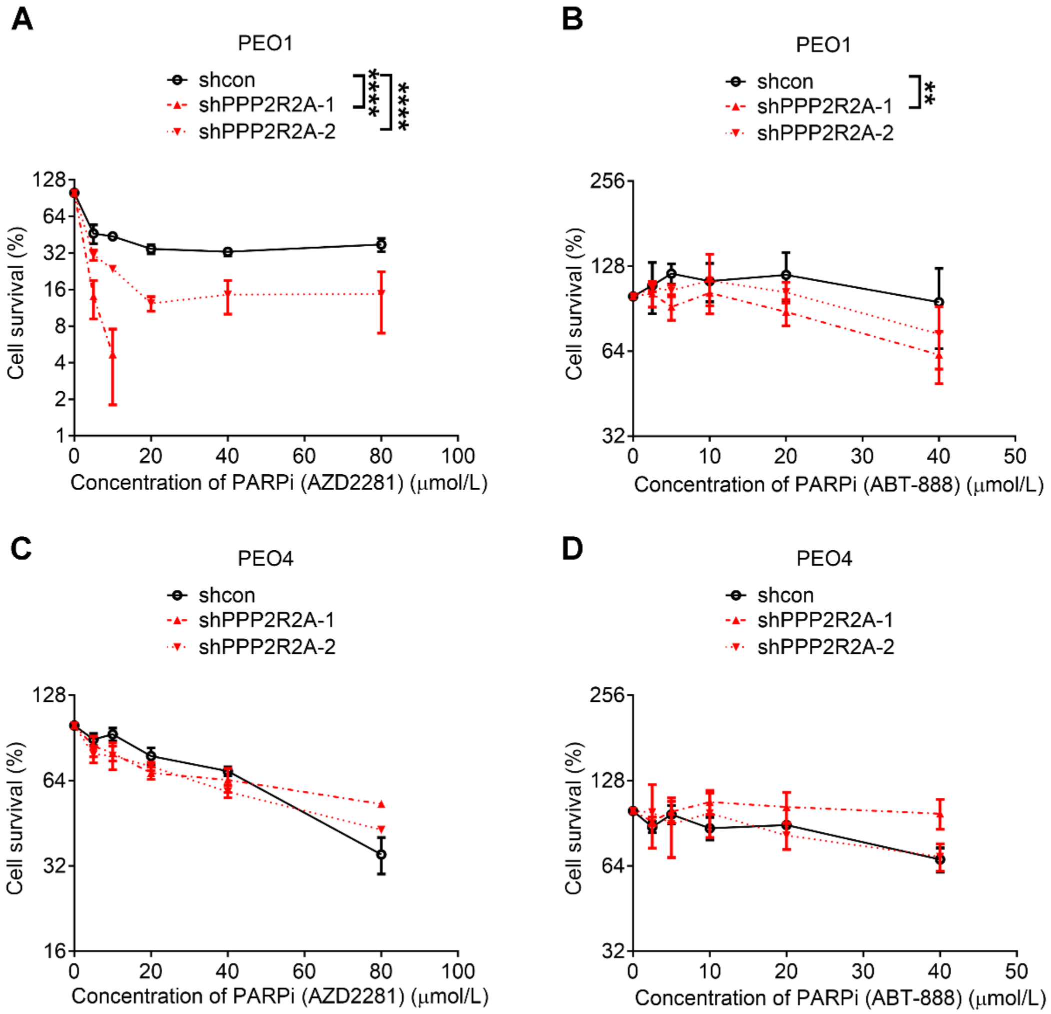


**Figure S4**. PEO1 cells with low expression of *PPP2R2A* are synthetically lethal with PARP inhibitors. Cellular toxicity to measure cell survival after treatment with PARP inhibitors AZD2281 (**A, C**) and ABT-888 (**B**, **D**) in PEO1 and PEO4 cells. n=3, biological repeats. The treatment time for AZD2281 and ABT-888 was 48 h. **, $P < 0.01$, ****, $P < 0.0001$, two-way ANOVA, followed by Bonferroni post hoc analysis for multiple comparisons was used to determine statistical significance.

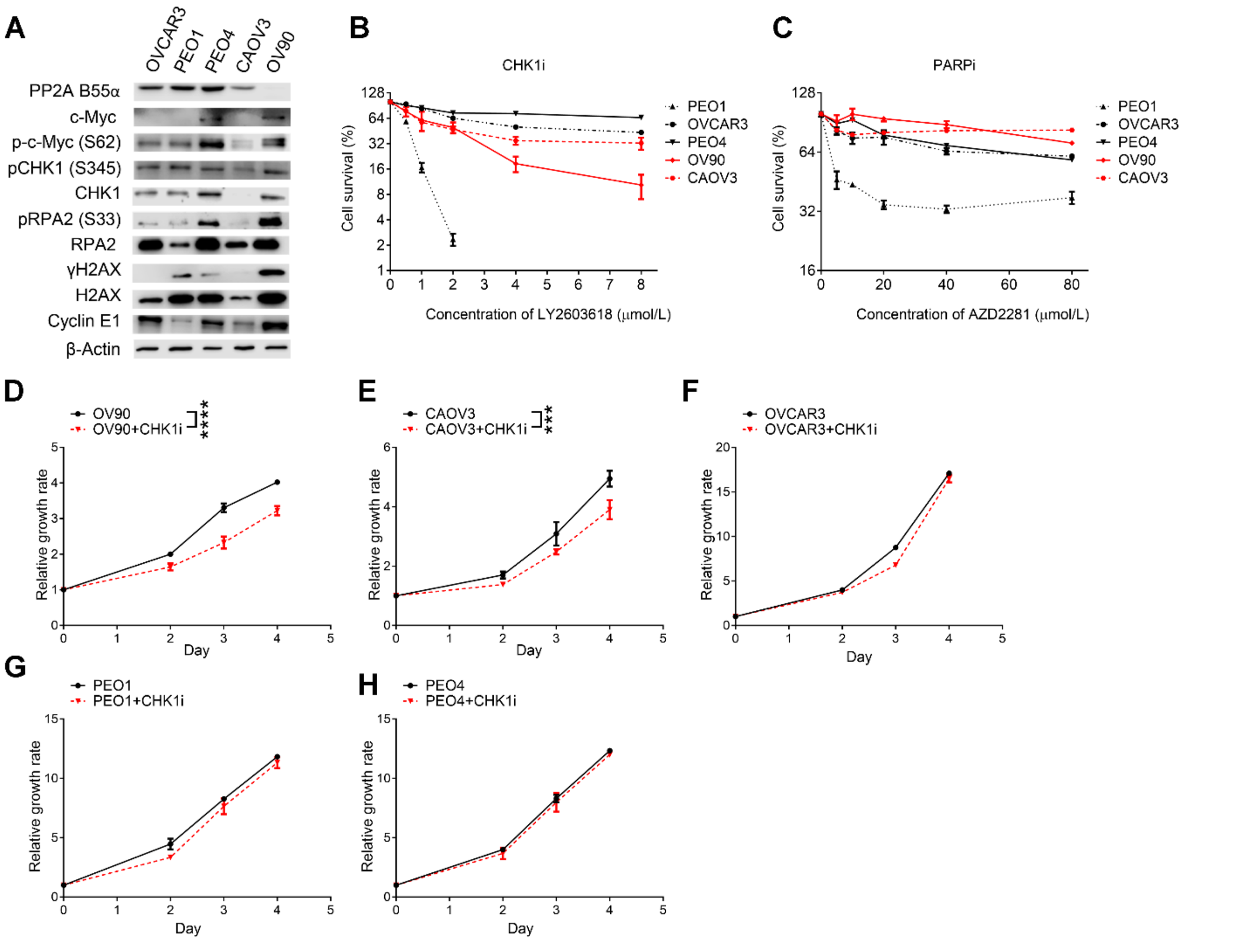


**Figure S5**. **CHK1 inhibition sensitized HGSOC cells with spontaneous low expression of *PPP2R2A***. (**A**) OV90 and CAOV3 cells have low expression of *PPP2R2A*. (**B-H**) HGSOC cells with low expression of PP2A B55α are more sensitive to CHK1 inhibition. PEO1 cells, which have BRCA2 mutation, exhibit sensitivity to both CHK1 and PARP inhibitors. MTT assays for the sensitivity CHK1 inhibitor LY2603618 (**B**), PARP inhibitor AZD2281 (**C**) in 5 human ovarian cancer cells. The incubation time for the CHK1 inhibitor LY260361 and PARP inhibitor AZD2281 were 48 h. $n$ = 3, biological repeats (**B, C**). (**D-H**) Treatment with the CHK1 inhibitor LY2603618 (1 μmol/L for OV90, CAOV3, OVCAR3 and PEO4, 0.5 μmol/L for PEO1) suppressed the proliferation of OV90 (**D**) and CAOV3 (**E**) cells but did not affect the proliferation of OVCAR3 (**F**), PEO1 (**G**), and PEO4 (**H**) cells. $n$ = 3, biological repeats. ***, $P < 0.001$, ****, $P < 0.0001$, two-way ANOVA, followed by Bonferroni post hoc analysis for multiple comparisons was used to determine statistical significance.

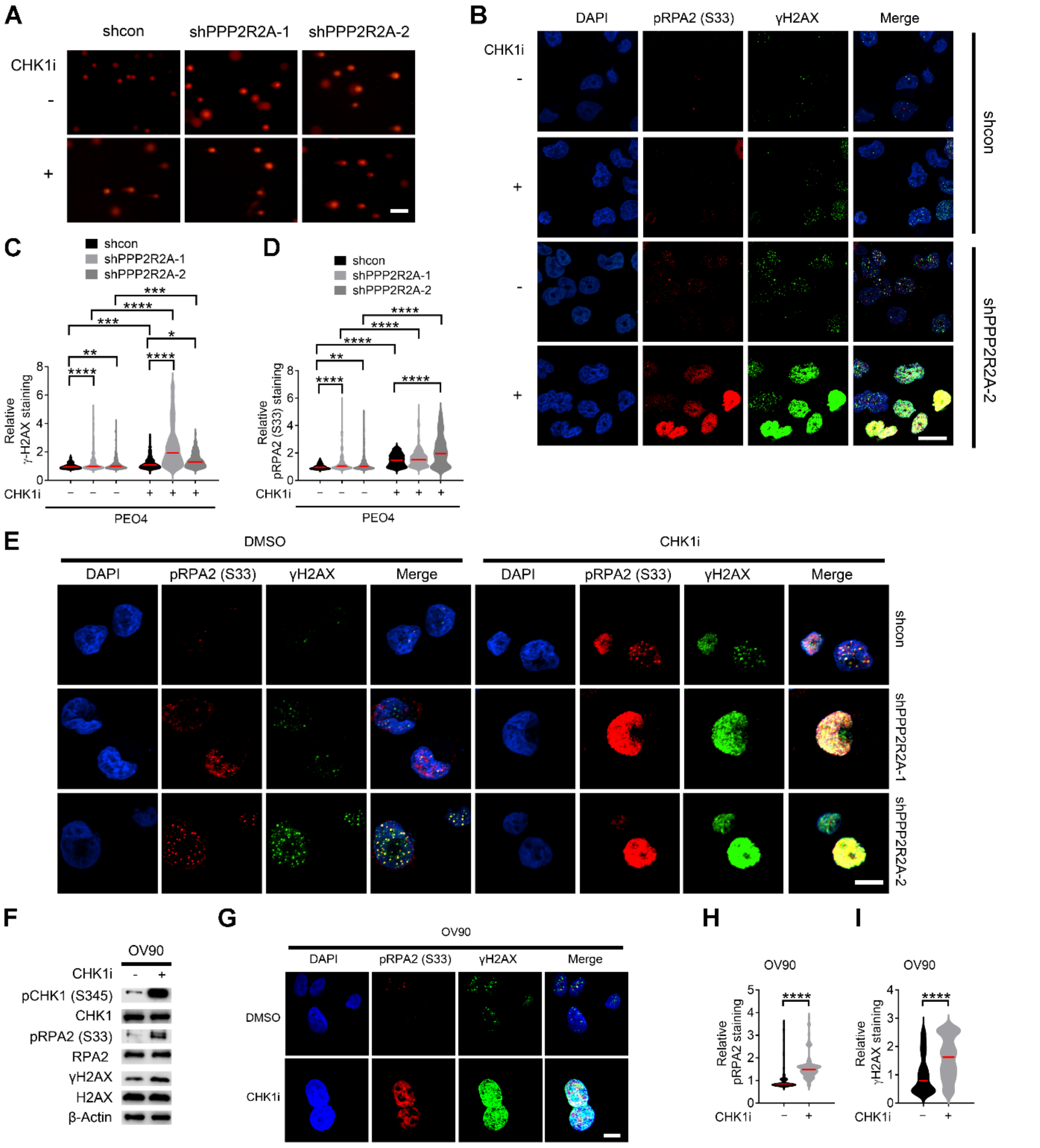


**Figure S6**. **CHK1 inhibition exacerbates RS, especially in *PPP2R2A* KD/deficient HGSOC cells**. (**A**) Representative images used for the quantification of olive tail moment in OVCAR3 cells. Scale bar, 40 μm. (**B**) Representative pictures of replication stress markers in OVCAR3 cells with or without *PPP2R2A* KD in the presence or absence of CHK1 inhibition. Scale bar, 20 μm. CHK1 inhibitor leads to enhanced staining density of replication stress markers, particularly in *PPP2R2A* KD PEO4 cells. (**C-E**). The staining density of γH2AX and p-RPA2 S33 (**C, D**) in PEO4 cells with or without *PPP2R2A* KD using immunofluorescence assay. Representative imaging of γH2AX and pRPA2 staining in PEO4 cells (**E**). Scale bar, 20 μm. Data in **C-D** are the

mean ± SEM of three independent experiments. n=300 in **C**-**D,** individual staining. Statistical significance was determined by one-way ANOVA, followed by Bonferroni post hoc analysis for multiple comparisons. *, $P < 0.05$; **, $P < 0.01$; ***, $P < 0.001$; ****, $P < 0.0001$. (**F**-**I**) RS markers increased in OV90 cells after CHK1 inhibition byLY2603618 (1 μmol/L) for 2 h. CHK1 inhibitor treatment upregulates the expression of pRPA2 S33, pCHK1 and γH2AX in OV90 cells (**F**). CHK1i leads to increased RS maker staining density in OV 90 cells (**G**). Scale bar, 10 μm. The staining density of γH2AX and pRPA2 are shown in (**H, I**). *n* = 150 in **G**, **H,** individual staining. Statistical significance was determined by Student's *t*-test. ****, $P < 0.0001$.

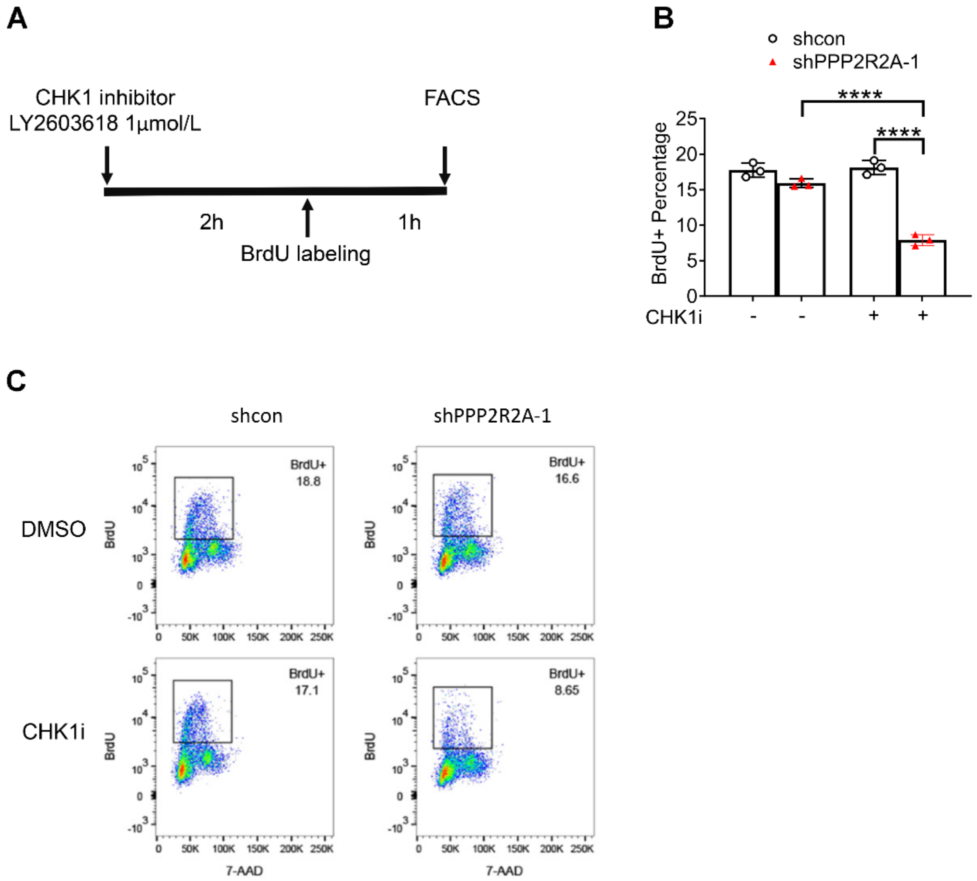


**Figure S7**. **CHK1 inhibition results in the reduction of percentage of BrdU positive cells in HGSOC cells with *PPP2R2A* KD.** (**A**) OVCAR3 cells, with or without *PPP2R2A* KD, were treated with CHK1 inhibitor for 2 h, followed by BrdU pulse labeling and FACS analysis. (**B**) CHK1 inhibition decreases the percentage of cells in the S phase, particularly in the cells with *PPP2R2A* KD, as indicated by BrdU incorporation. Representative figures of BrdU labeling are shown in (**C**). Data represent the mean ± SEM of three biological repeats (n=3). ****, $P < 0.0001$, one-way ANOVA, followed by Bonferroni post hoc analysis for multiple comparisons was used to determine statistical significance.

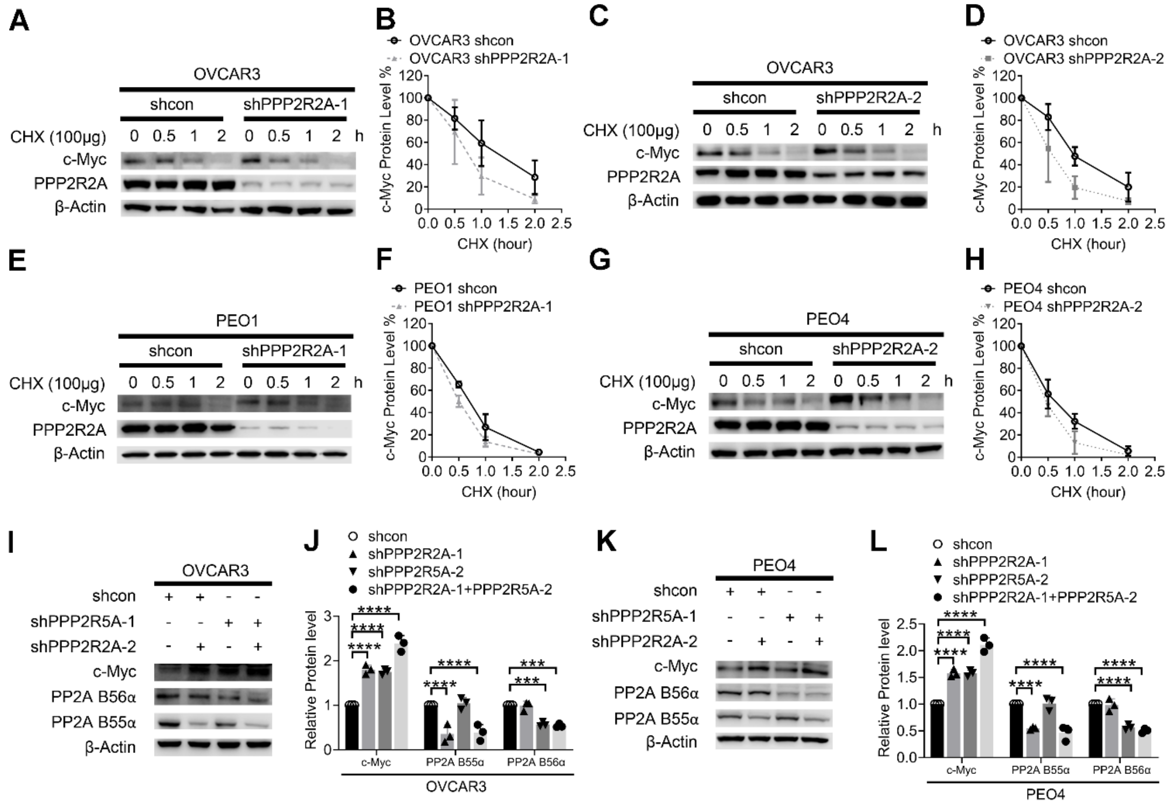


**Figure S8**.**c-Myc upregulation in *PPP2R2A* KD HGSOC cells is independent of regulation of c-Myc degradation and PP2A B56α**. The elevation in c-Myc protein is independent of its degradation in *PPP2R2A* KD HGSOC cells (**A**, **C**, **E** and **G**). The protein degradation of c-Myc was analyzed in OVCAR3 (**B**, **D**), PEO1 (**F**) and PEO4 (**H**) cells treated with cycloheximide (100 μg/ml). The protein degradation rates of c-Myc were determined by biological repeats ($n$ = 3, **B**, **D, F** and **H**). *PPP2R2A* KD elevates c-Myc protein in PP2A B56α stable knockdown OVCAR3 (**I**, **J**) and PEO4 cells (**K**, **L**). $n$ = 3 in **J** and **L,** biological repeats. Statistical significance was determined by one-way ANOVA, followed by Bonferroni post hoc analysis for multiple comparisons. ***, $P$ < 0.001; ****, $P$ < 0.0001.

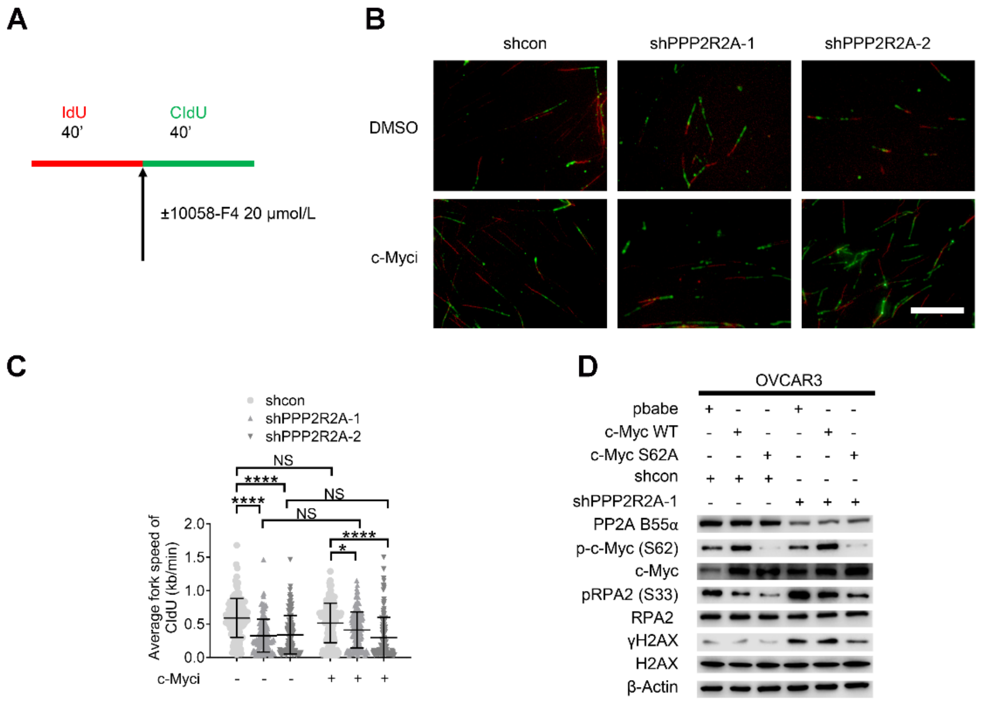


**Figure S9**. **c-Myc inhibition abrogates *PPP2R2A* KD-induced replication stress in OVCAR3 cells.** DNA fiber results of c-Myc inhibition in OVCAR3 cells with or without PPP2R2A knockdown (**A**-**C**)**.** (**A**) A schematic diagram of the labeling scheme in OVCAR3 cells for c-Myc inhibition: IdU is incorporated as the first labeling for 40 min, followed by incorporation of CldU as the second labeling plus c-Myc inhibitor treatment for 40 min. (**B**) Representative images of DNA fibers from OVCAR3 cells treated with c-Myc inhibitor 10058-F4 (20 µmol/L) for 2 h. Scale bar, 100 µm. c-Myc inhibition does not change the of CldU fork speed. The average fork speed of CldU labelling is shown in (**C**). n=300 in **C,** individual counting of each fiber. Data in **C** are the mean ± SEM of three independent experiments. Statistical significance was determined by one-way ANOVA, followed by Bonferroni post-hoc analysis for multiple comparisons. ***, $P <$ 0.001; ****, $P < 0.0001$. (**D**) Stable over expression of c-Myc S62A mutant in OVCAR3 cells abolishes *PPP2R2A* KD-induced pRPA2 and γH2AX expression.

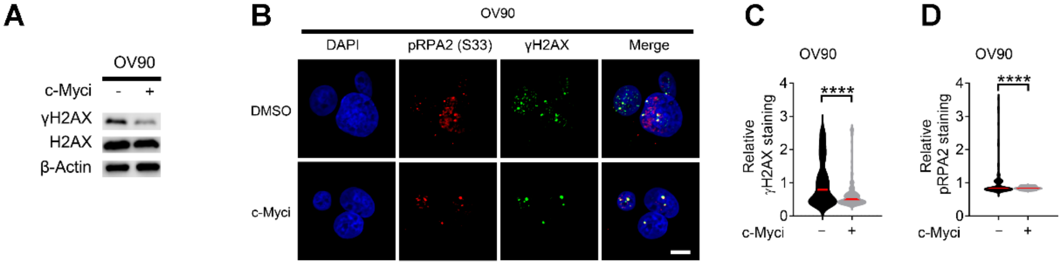


**Figure S10**. **c-Myc inhibition decreases the RS in HGSOC cells with low expression of *PPP2R2A*.** (**A-D**) Replication stress markers decrease in OV90 cells after treatment of c-Myc inhibitor 10058-F4 (20 μmol/L) for 2 h. c-Myc inhibition decreases the expression of pCHK2 and γH2AX in OV90 cells (**A**). c-Myci leads to decreased RS maker staining density in OV 90 cells (**B**). Scale bar, 10 μm. The staining density of γH2AX and pRPA2 are shown in (**C, D**). *n* = 150 in **C**, **D,** individual staining. Statistical significance was determined by Student's *t*-test. ****, $P <$ 0.0001.

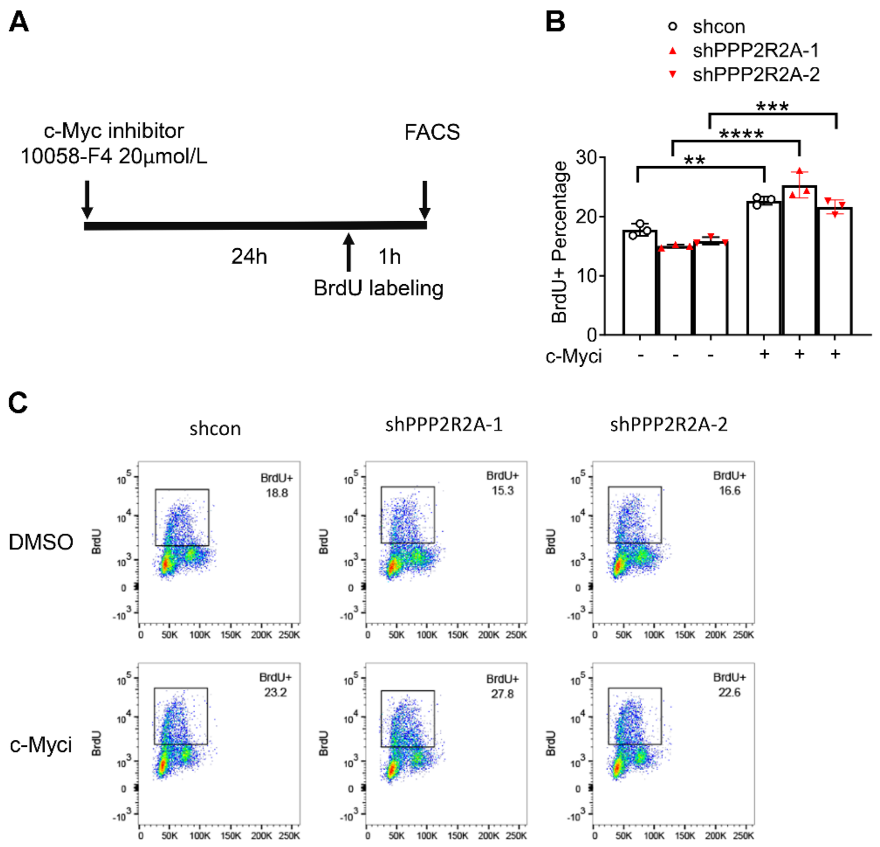


**Figure S11**. **c-Myc inhibition leads to the S phase arrest in HGSOC cells with *PPP2R2A* KD.** (**A**) OVCAR3 cells were treated with c-Myc inhibitor for 24 h, followed by BrdU pulse labeling and FACS analysis. (**B**) c-Myc inhibition increases the percentage of cells in the S phase, as indicated by BrdU incorporation. Representative figures of BrdU labeling are shown in (**C**). Data represent the mean ± SEM of three biological repeats ($n$ = 3). *, $P < 0.05$; **, $P < 0.01$; ***, $P < 0.001$; ****, $P < 0.0001$, one-way ANOVA, followed by Bonferroni post-hoc analysis for multiple comparisons was used to determine statistical significance.

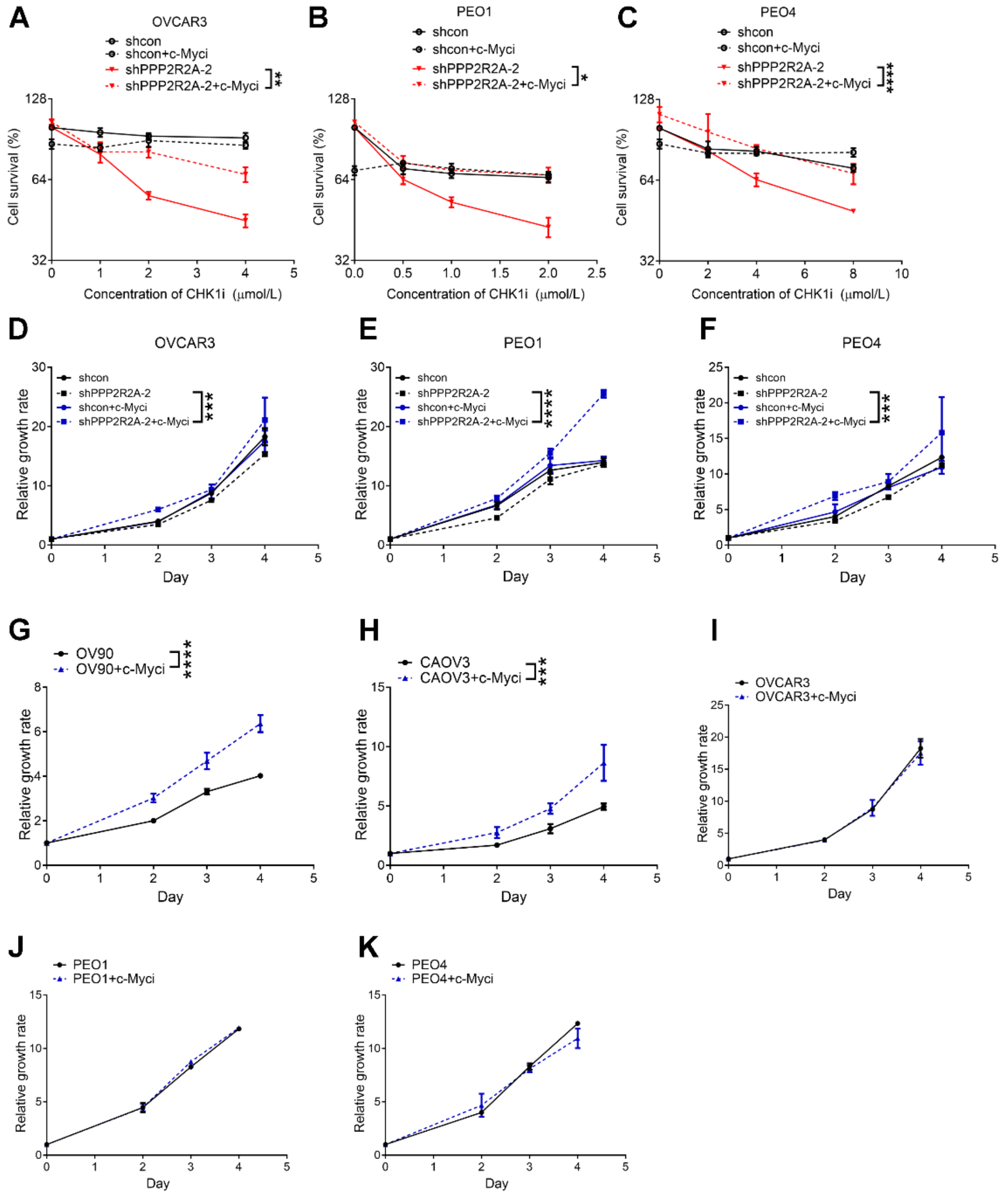


**Figure S12**. **c-Myc inhibition reduces *PPP2R2A* KD/deficiency-induced sensitivity to CHK1 inhibition.** (**A-C**) c-Myc inhibition decreases *PPP2R2A* KD-triggered sensitivity to CHK1 inhibition in OVCAR3 (**A**), PEO1 (**B**) and PEO4 (**C**) cells. (**D-K**) Treatment with a c-Myc inhibitor (20 μmol/L) leads to increased cellular growth in *PPP2R2A* knockdown OVCAR3 cells (**D**), PEO1 cells (**E**), and PEO4 cells (**F**). Additionally, c-Myc inhibition increased the proliferation of OV90 (**G**) and CAOV3 (**H**) cells, both of which have low expression of PPP2R2A. However, c-Myc inhibition did not enhance the proliferation of OVCAR3 (**I**), PEO1 (**J**), and PEO4 (**K**) cells, which have high expression of PPP2R2A. $n$ = 3, biological repeats. *, $P < 0.05$; **, $P < 0.01$; ***, $P < 0.001$; ****, $P < 0.0001$, two-way ANOVA, followed by Bonferroni post-hoc analysis for multiple comparisons was used to determine statistical significance.
